# Effects of ketamine optical isomers, psilocybin, psilocin and norpsilocin on time estimation and cognition in rats

**DOI:** 10.1007/s00213-021-06020-5

**Published:** 2022-03-02

**Authors:** Piotr Popik, Adam Hogendorf, Ryszard Bugno, Shaun Yon-Seng Khoo, Pawel Zajdel, Natalia Malikowska-Racia, Agnieszka Nikiforuk, Joanna Golebiowska

**Affiliations:** 1 Behavioral Neuroscience and Drug Development, Maj Institute of Pharmacology, Polish Academy of Sciences, Smętna 12, 31-343 Kraków, Poland; 2 Medicinal Chemistry, Maj Institute of Pharmacology, Polish Academy of Sciences, Smętna 12, 31-343 Kraków, Poland; 3 Department of Pharmacology and Physiology, Faculty of Medicine, Université de Montréal, Montreal, QC Canada; 4 Department of Organic Chemistry, Jagiellonian University Medical College, Medyczna 9, 30-383 Kraków, Poland

**Keywords:** Time perception, Cognition, Ketamine, Psilocybin, Rapid antidepressant, Rat

## Abstract

**Rationale:**

Ketamine and psilocybin belong to the rapid-acting antidepressants but they also produce psychotomimetic effects including timing distortion. It is currently debatable whether these are essential for their therapeutic actions. As depressed patients report that the “time is dragging,” we hypothesized that ketamine and psilocybin-like compounds may produce an opposite effect, i.e., time underestimation, purportedly contributing to their therapeutic properties.

**Objectives:**

Timing was tested following administration of (*R*)- and (*S*)-ketamine, and psilocybin, psilocin, and norpsilocin in the discrete-trial temporal discrimination task (TDT) in male rats. Timing related to premature responses, and cognitive and unspecific effects of compounds were tested in the 5-choice serial reaction time task (5-CSRTT) in the standard 1-s, and “easier” 2-s stimulus duration conditions, as well as in the vITI variant promoting impulsive responses.

**Results:**

(*S*)-ketamine (15 but not 3.75 or 7.5 mg/kg) shifted psychometric curve to the right in TDT and reduced premature responses in 5-CSRTT, suggesting expected time underestimation, but it also decreased the accuracy of temporal discrimination and increased response and reward latencies, decreased correct responses, and increased incorrect responses. While (*R*)-ketamine did not affect timing and produced no unspecific actions, it reduced incorrect responses in TDT and increased accuracy in 5-CSRTT, suggesting pro-cognitive effects. Psilocin and psilocybin produced mainly unspecific effects in both tasks, while norpsilocin showed no effects.

**Conclusions:**

Time underestimation produced by (*S*)-ketamine could be associated with its antidepressant effects; however, it was accompanied with severe behavioral disruption. We also hypothesize that behavioral disruption produced by psychedelics objectively reflects their psychotomimetic-like actions.

**Supplementary Information:**

The online version contains supplementary material available at 10.1007/s00213-021-06020-5. MED-PC code for TDT and the raw data are available at 10.6084/m9.figshare.14933127.

There is growing interest in medications that produce immediate relief of the symptoms of depression. This emerging class of compounds has been named as “rapid-acting antidepressants” (Krystal 2007) and includes glutamate NMDA receptor antagonist ketamine, anti-muscarinic scopolamine, and serotonin (5-HT)_2A/1A_ receptor agonist psilocybin (Witkin et al. 2018).

The idea that NMDA receptor antagonists could be useful in the treatment of affective disorders came from the seminal work of Trullas and Skolnick (1990) at the NIH, demonstrating that antagonism of NMDA receptors produces antidepressant phenotypes in rodents (see Skolnick et al. (2009) for a review). For the NMDA receptor antagonist ketamine (Zukin et al. 1984), first Berman et al. (2000) and then Zarate Jr et al. (2006) reported that treatment-resistant depressed patients began to demonstrate an antidepressant response within hours of drug administration. Importantly, ketamine administration in these patients was associated with transient cognitive impairments and perceptual changes consistent with psychotomimetic effects of ketamine observed in healthy individuals (Krystal et al. 1994; Pomarol-Clotet et al. 2006).

Not counting for its metabolites, ketamine exists in two chiral forms. In 2019, the (*S*)-ketamine enantiomer (Esketamine ®) was approved for use in treatment-resistant depression in the USA (Spravato ®) and then in Europe (Cristea and Naudet 2019). However, recent data suggest differences in the pharmacological and subjective, dissociative effects between ketamine enantiomers. For instance, as compared with (*R*)-*(-)*-ketamine, (*S*)*-(+)*-ketamine binds with a 3–4 time higher affinity to the PCP binding site of the NMDA receptor (Zukin et al. 1984) and affects opioid neurotransmission (Bonaventura et al. 2021; Finck and Ngai 1982). This is likely responsible for stronger psychotomimetic effects of the (*S*)-isomer. In support, Vollenweider et al. (1997) found (*S*)-ketamine produced psychotic-like effects whereas comparable doses of (*R*)-ketamine produced a state of relaxation, elation, and calmness. In agreement, Persson et al. (2002) reported that (*R*)-ketamine induced less subjective effects than (*S*)-ketamine. A recent study demonstrated that (*R*)-ketamine produces antidepressant effects at doses engendering relatively benign side-effect profile (Leal et al. 2020). Interestingly, also in this study, all (*R*)-ketamine-treated patients reported the sensations of serenity and inner peace.

Antidepressant effects of psilocybin were shown in two randomized, double-blind, crossover design studies in patients with life-threatening cancer. A substantial and sustained decrease in depression was demonstrated (Griffiths et al. 2016; Ross et al. 2016). Similar effects were reported for psilocybin in the patients with treatment-resistant depression in an open-label feasibility study (Carhart-Harris et al. 2016). Interestingly, the therapeutic outcome was associated with a “mystical” subjective experience. However, animal studies failed to detect an antidepressant-like effects of psilocybin (Jefsen et al. 2019).

Could the psychotic-like, dissociative-like, hallucinogenic-like, mystical, and/or psychotomimetic-like experiences be responsible for the therapeutic, i.e., antidepressant actions of ketamine and psilocybin? While in case of ketamine this issue is debatable (Ballard and Zarate Jr. 2020), it is likely that profound perceptual experience can act therapeutically. Psychedelics have been used in the therapy of psychiatric disorders (e.g., ibogaine in the treatment of addictive disorder; see Popik et al. (1995) for a review). If this is so, the question remains as to the nature of subjective experience produced by ketamine and psilocybin and whether at all, it could be measured in infra-human animals.

One of the distortions produced by psychotomimetics that could be measured in animals implies their effects on time perception. Ketamine alters experience of time in healthy volunteers (Pomarol-Clotet et al. 2006) in the form of slowing, though some participants report an opposite increase in subjective rate. However, in rats, racemic (*R,S*)-ketamine at a single dose of 15 mg/kg did not affect timing (Cheng et al. 2006). Regarding psilocybin, one human study demonstrated selective disruption of timing at longer intervals (Wittmann et al. 2007). The authors interpreted this effect as a product of interactions with cognitive dimensions of temporal processing, rather than of interactions with the basic pacemaker/accumulator mechanisms of the brain (Meck 1983, 1997), likely reflecting impairments of short-term memory, attention, or decision-making mechanisms.

We hypothesized that ketamine isomers and psilocybin-like compounds may produce time underestimation, purportedly contributing to their therapeutic properties. This is because depressed patients experience timing distortion and report that the “*time is dragging*,” and when tested, they overestimate the cues of long durations (Caceda et al. 2020); see “Discussion.” We thus attempted to replicate Cheng et al. (2006) findings with (*R,S*)-ketamine in the temporal discrimination task (Killeen et al. 1997) using (*S*)- and (*R*)-ketamine enantiomers as they produce different subjective effects in humans (see above). We also investigated the effects of psilocybin as well as its metabolite psilocin, and the novel psilocybin derivative, norpsilocin (Sherwood et al. 2020); as to the best of our knowledge, their effects on timing in rats have not been reported.

The results of present studies revealed that only (*S*)-ketamine produced time underestimation, an effect that was, in addition, accompanied by numerous unspecific effects, while other compounds were either inactive or produced only unspecific effects. To get a better understanding of the nature of the unspecific effects of psychedelics in TDT, and because premature responses were related to timing (Cope et al. 2016), we used 5-choice serial reaction time task (5-CSRTT), which is a food-motivated attention test analogous to the continuous performance task used to study attention in humans (Robbins 2002). 5-CSRTT is well-suited to characterize the effects of psychotropic drugs because it yields metrics that quantify attention, reaction time, motivation, and impulsivity (Paine et al. 2007; Robbins 2002). Several laboratories (Benn and Robinson 2014; Gastambide et al. 2013; Higgins et al. 2021; Nemeth et al. 2010; Oliver et al. 2009; Smith et al. 2011), including ours (Nikiforuk and Popik 2014), have already examined the effects of ketamine racemate in this task, but a direct comparison of (*R*)- and (*S*)-ketamine has never been reported. The effects of psilocybin in the 5-CSRTT have been recently reported by Higgins et al. (2021).

## Methods

### Animals

Twenty (TDT) and 40 (5-CSRTT) experimentally naïve male Sprague-Dawley rats (Charles River, Germany) weighing ~250 g at the arrival were group housed 5 per cage in the standard laboratory cages, under standard colony A/C controlled conditions: room temperature 21 ± 2 °C, humidity (40–50%), and 12-h light/dark cycle (lights on: 06:00). The animals were kept with ad libitum access to water and with a mild food restriction (~17–20 g of food per day) for at least 1 week prior to training and were maintained at 85% of their free-feeding weight throughout the study. All animals were maintained, and experiments were conducted in accordance with the NIH Guide for the Care and Use of Laboratory Animals and approved by the II Local Ethics Committee for Animal Experiments at the Maj Institute of Pharmacology, Polish Academy of Sciences, Kraków, Poland.

### Apparatus

For the temporal discrimination task, 4 operant chambers (Med Associates, St. Albans, VT, USA) measuring 56 cm × 56 cm × 40.5 cm were housed in sound-attenuated and ventilated cubicles. In each chamber, the food magazine, which was equipped with photocell beams and light, was located between two retractable levers. A house light (5-W white bulb) was located 17 cm above the top edge of the food magazine. Food pellets (45 mg, #F0165, Bio-Serv Dustless Precision Pellets, Frenchtown, NJ, USA) were delivered via a dispenser connected to the food magazine. Online control of the apparatus and data collection was performed using MED-PC. The exhaust fan provided continuous masking noise.

The construction of 8 operant boxes for the 5-CSRTT was similar; however, there were no retractable levers but instead, the wall opposite to the food magazine contained 5 LED-illuminated nose-poke operandi (Nikiforuk and Popik 2014).

### Temporal discrimination task (TDT) procedure

The procedure was based on several earlier protocols (Bizot 1997; Halberstadt et al. 2016; Hampson et al. 2010; Maricq et al. 1981). Med-PC code for the TDT as well as the raw data are available at 10.6084/m9.figshare.14933127.

#### Magazine training

During the initial training phase, the rats had to learn that food pellets were available in the magazine. On the first day, the rats were habituated to the operant chambers for 20 min. During this habituation session, the food magazine was filled with several pellets. Next, rats were given magazine training sessions in which every head entry into a food magazine resulted in a pellet delivery. Once all the rats consumed 80 food pellets within a session (which took two sessions), the training proceeded to the next stage.

#### Pre-training

In this phase, the rats had to associate a lever press with a pellet delivery. Either the left or the right lever was available and every lever press was reinforced with a food pellet (six 20-min sessions). Thereafter, the animals were subjected to five 20-min sessions with both levers presented. Pressing either lever resulted in the immediate withdrawal of the lever and the delivery of one food pellet. There was a 10-s inter-trial interval (ITI) between trials.

#### Training sessions

Fifteen seconds after insertion of the rat into the box, the magazine light was turned on (Supplemental Figure 1). Every trial was initiated by the illumination of the stimulus house light (Maricq et al. 1981) for either 3 s (short stimulus) or 12 s (long stimulus). When the house light was extinguished, two levers were inserted into the chamber. If no response occurred within 10 s after the insertion of the levers, they were retracted, the magazine light was turned off, and a *response omission* was recorded. Lever withdrawal initiated an ITI of 10 s. A response on either lever resulted in the immediate withdrawal of both levers. During trials in which the 3-s stimulus was presented, a response on lever A (*correct response*) resulted in the lever retraction and the delivery of one food pellet, whereas a response on lever B (*incorrect response*) resulted in the lever retraction and turning off the magazine light. Conversely, in trials, in which the 12-s stimulus was presented, a response on lever B (*correct response*) was reinforced whereas pressing lever A (*incorrect response*) was not. The positions of levers A and B (left vs. right) were counterbalanced across subjects. For all animals, the lever assignments remained unchanged throughout the study.

Every training session consisted of 40 trials with 3-s stimulus and 40 trials with 12-s stimulus; the ratio of stimuli presentations was 0.5 and the sequence stimuli presentations was randomized. Ten percent of the trials were forced choice trials where only one lever was extended and was not retracted until a response was made (Halberstadt et al. 2016; Hampson et al. 2010).

#### Testing sessions

Training occurred until all animals attained 85% correct responses for the 3- and 12-s stimuli (2 weeks, data not shown). Testing sessions were identical to the training sessions except that besides using the 3- and 12-s anchor stimuli, also intermediate durations of stimuli (4, 5, 6, 7, 8, 9, 10, and 11 s) were introduced. There were no forced choice trials on the testing sessions. In the testing session, there were 5 presentations of every stimulus (with 10 stimuli, 50 trials total) and all the stimuli were presented in a random order. Responding on lever A was reinforced if the stimulus was ≤ 7 s and responding on lever B was reinforced if the stimulus was ≥ 8 s (Halberstadt et al. 2016; Hampson et al. 2010). Correct and incorrect responses as well as omissions (Supplemental Figure 1) were recorded.

### Temporal discrimination task (TDT) experimental design and statistics

Rats were subjected to daily sessions 5 days per week (Monday–Friday) at the same time each day, during the light phase of their light-dark cycle. Testing sessions were randomized with respect to the treatments, were done once or twice a week, and were always preceded by at least two training sessions or a no testing day and a training session. Every treatment group had its own vehicle control. At the testing session, every compound’s dose (or the vehicle) was administered to all animals. The sequence of drug testing is presented in Table 1. Since the highest doses of ketamine enantiomers were tested at the very end of the study, the risk of a carry-over effect was minimized.

**Table 1:** Sequence of drug testing in the temporal discrimination task (TDT)

Week(s) of testing	Treatment
1–4	“Low” doses of (*R*)-ketamine (0, 7.5, 15 mg/kg)
	“Low” doses of (*S*)-ketamine (0, 3.5, 7.5 mg/kg)
5–6	Wash-out and training with vehicle
7–8	Psilocybin (0, 0.3, 1 mg/kg)
8–9	Wash-out and training with vehicle
9–10	Psilocin (0, 0.22, 0.72 mg/kg)
11–12	Wash-out and training with vehicle
13–14	Norpsilocin (0, 0.22, 0.72 mg/kg)
15	Wash-out and training with vehicle
16–18	“High” doses of (*R*)-ketamine (0, 30 mg/kg)
	“High” doses of (*S*)-ketamine (0, 15 mg/kg)

#### Quantitative analysis of individual psychometric functions

For every treatment condition and every animal in that treatment condition, we first examined omissions, and data of animals showing ≥ 40% of omissions for any stimulus duration were eliminated (Bizot 1997). Quantitative analysis of individual rat psychometric functions was performed using GraphPad Prism’s nonlinear regression 2-parameter logistic function *f*(*x*)=1/(1+exp((-eta*(*t*-T_50_)))) (Gouvea et al. 2015) with automatic outlier elimination. In this equation, *t* is the stimulus duration, T_50_ is the stimulus duration corresponding to %*B* = 50% (sometimes called PSE; point of subjective equality) and eta (*ε*) is the slope of the function. As in Halberstadt et al. (2016), we excluded from analyses the observations that logistic function failed to fit the %*B* responding data. Besides the T_50_ and slope function data, the individual curve-fitting procedure yields estimates of the values of T_25_ and T_75_ from which the difference limen (“just noticeable difference”; JND) and Weber fraction were determined as in Hampson et al. (2010). The limen was defined as half the difference between T_75_ and T_25_ and the Weber fraction was calculated as the ratio of the difference limen to T_50_. These two estimates indicate the accuracy of discrimination.

#### Qualitative analysis of various parameters

The Weber fraction, difference limen, slope, and T_50_ data were analyzed by one-way ANOVA or—in case of not normal distribution—the Kruskal-Wallis test followed by Dunnet’s or Dunn’s post hoc tests, respectively. The proportion of choices of the 12-s B lever was analyzed by two-way mixed design ANOVA with treatment and stimulus duration as the factors, followed by the post hoc LSD test (Cardinal and Aitken 2006). For every animal within given treatment condition and every stimulus duration, we also analyzed the response latency, i.e., the time that passed from lever availability to the correct lever press. This is because the horizontal psychometric curve shift could be due to the time over/underestimation but it also could be due to a change in response latency (Maricq et al. 1981). In addition, we analyzed the reward latencies, i.e., the time that passed between correct lever press and magazine visit. While the response latency addresses the speed of cognitive processing (see Amitai and Markou (2010) for a review), the reward latency measures general motor function and addresses motivational factors. This approach, not common in TDT studies, was taken from analyses of cognitive functioning in the 5-CSRTT, in which ketamine increases response latency (see Fig 2 in Nikiforuk and Popik (2014)). The latency data of individual animals were first averaged for a given stimulus duration, because during the test there could be as many as 5 trials of a given stimulus duration, see above. Thus, with stimuli of 10 different durations, every animal/test latency data are represented as a set of 10 averaged responses. Lastly, we analyzed the proportion of response omissions, and correct, and incorrect responses. The latter measures as well as the latencies were also analyzed by two-way ANOVA.

Statistical analyses were done with IBM SPSS ver. 26 and GraphPad Prism; Levene’s test of equality of error variances was used throughout. The alpha level was set at 0.05.

### 5-choice serial reaction time task (5-CSRTT) procedure

The beginning of each session was signaled with the house light illumination and food pellet delivery. To initiate the first trial, rat had to nose-poke into a food magazine. Each trial consisted of an ITI (inter-trial interval) followed by the random illumination of one of the five holes for a fixed interval (stimulus duration, Sd). The rat had to respond within the limited hold (Lho). Every correct response was rewarded and every incorrect response or a failure to respond within the required period (omission) resulted in a time out (TO) period (house light was extinguished). Premature responses (nose-poke during the ITI) also resulted in a TO.

In the training sessions, rats had to learn that correct responding to an illuminated nose-poke results in a food reward. Animals were trained in changing conditions, starting with the primary parameters to final parameters in a basic 5-CSRTT protocol as in (Nikiforuk and Popik 2014). The final 5-CSRTT test parameters in the basic protocol were as follows: Sd = 1 s, Lho = 5 s, ITI = 5 s, TO = 5 s. Each session consisted of 100 trials or lasted up to 60 min. Rats were qualified for the test after reaching the following criteria: accuracy > 70%, omissions < 30%, and stable baseline performance across 3 consecutive sessions.

Animals were tested in 3 variants of the 5-CSRTT: (i) the basic protocol with the test parameters same as in training phase (standard conditions with Sd = 1 s); (ii) in a variant with extended duration of the stimulus (Sd = 2 s; “easier” conditions), and (iii) in variable ITI (vITI), in which every session consisted of 100 trails with 1/4 of the trials of ITIs of 2.5, 5, 7.5, and 10 s. Testing sessions were randomized with respect to the treatments, were done once or twice a week, and were always preceded by at least two training sessions. Every treatment group had its own vehicle control. Animals demonstrating low performance on the training session preceding the test were excluded from statistical analyses.

### 5-CSRTT study experimental design and statistics

The order of 5-CSRTT experiments, including the week of testing, compounds, their doses, and the *N* of rats tested, is shown in Table 2.

**Table 2:** Experimental design of the 5-choice serial reaction time task (5-CSRTT) study

Week of testing	5-CSRTT variant		Treatment	Dose mg/kg (*N*)	Treatment	Dose mg/kg (*N*)	
1	I	SD = 1 s	(*R*)-Ketamine	veh (12) 7.5 (13) 15 (13)	(*S*)-Ketamine	veh (13) 3.75 (13) 7.5 (12)	
2	I	SD = 1 s					
5	II	SD = 2 s		veh (12) 7.5 (13) 15 (13)		veh (12) 3.75 (13) 7.5 (13)	
6	II	SD = 2 s					
7	III	Variable ITI		veh (13) 7.5 (13) 15 (13)		veh (12) 3.75 (13) 7.5 (13)	
8	III	Variable ITI					
9	I	SD = 1 s	Psilocybin	veh (13); 0.3 (13); 1 (8)			
11	I	SD = 1 s	Psilocin	veh (14); 0.22 (13); 0.72 (8)			
14	I	SD = 1 s	Norpsilocin	veh (12); 0.22 (13); 0.72 (13)			
16	I	SD = 1 s	(*R*)-Ketamine	veh (12) 30 (15)	(*S*)-Ketamine	veh (13) 15 (14)	
17	I	SD = 1 s					
18	II	SD = 2 s		veh (13) 30 (15)		veh (13) 15 (15)	
18	II	SD = 2 s					
20	III	Variable ITI		veh (19) 30 (20)		veh (17) 15 (20)	
21	III	Variable ITI					
22	II	SD = 2 s	Psilocybin	veh (12); 0.3 (13); 1 (8)			
22	II	SD = 2 s	Psilocin	veh (11); 0.22 (13); 0.72 (10)			

The effects of (*R*)- and (*S*)-ketamine were tested in all three 5-CSRTT variants, psilocybin and psilocin were tested in the variants with Sd 1-s and 2-s, while norpsilocin that produced no effects in TDT and in the standard Sd 1-s 5-CSRTT variant was not further tested in Sd 2-s or vITI variants.

The following parameters were recorded in each session: percent *accuracy* (number of correct responses divided by the sum of correct and incorrect responses × 100), number of *omissions* (total number of trials omitted during a 100-trial session), *premature responses* (total number of responses performed during the ITIs), *perseverative responses* (total number of responses performed after a correct response, but before collection of the reward, divided by the total number of trials), *correct response latency* (time from the stimulus onset to a correct response), *incorrect response latency* (time from the stimulus onset to an incorrect response), and *reward latency* (time from a correct response to the retrieval of food from the magazine).

Statistical analyses of 5-CSRTT data were done with IBM SPSS ver. 26 and included 1- or 2-way ANOVAs, typically of a mixed design with the time as repeated measure and treatment as between-subjects factor, with the Sidak post hoc test (Cardinal and Aitken 2006). Wherever data were missing, as in the case of response omissions, 2-way between-subjects design was used, and in the cases of nonparametric distributions, the Kruskal-Wallis test followed by the Dunn’s post hoc tests was used. Levene’s test of equality of error variances was used throughout. The alpha level was set at 0.05.

### Drugs

(*R*)-ketamine HCl and (*S*)-ketamine HCl were separated and purified by AH from the racemate at the Department of Medicinal Chemistry of the Maj Institute of Pharmacology using the method described by Gao et al. (2020). The enantiomeric purity was determined by HPLC using ReproSil Chiral-OM 4.6 × 250 mm, 5-μm column (dr Maisch GMBH) using heptane:isopropanol 95:5 as eluent at 0.7 ml/min flow. The retention times were *t*_R_ = 12.12 min and *t*_R_ = 13.60 min for (*R*)- and (*S*)-ketamine, respectively. The obtained enantiomers were of ee > 99%. Ketamine enantiomers were dissolved in sterile water (vehicle:sterile 0.9% saline) prior to injection.

Psilocybin and psilocin were synthesized by AH and RB at the Department of Medicinal Chemistry of the Maj Institute of Pharmacology using the method described by Shirota et al. (2003). The tetrabenzyl pyrophosphate used was prepared according to the procedure described in Organic Syntheses by Nelson et al. (2003). Psilocin was additionally purified by filtration through a bed of celite and silicagel. Psilocybin and psilocin as well as norpsilocin (synthesized at the Department of Organic Chemistry, Jagiellonian University Medical College, following the method disclosed by Sherwood et al. (2020); compound purity > 99%) were dissolved in sterile distilled water acidified with 2 μl of glacial acetic acid (vehicle).

### Drug administration

All solutions were made fresh at the day of testing. The psilocybin compounds were kept in the vials protected from light under argon conditions. The doses of (*R*)- and (*S*)-ketamine were based on our earlier reports (Popik et al. 2020) and preliminary data (not shown). Assuming the (*S*)-isomer is at least twice as potent as (*R*)-ketamine at the NMDA receptors (Zukin et al. 1984), we used 7.5, 15, and 30 mg/kg of (*R*)*-*ketamine and 3.75, 7.5, and 15 mg/kg of (*S*)*-*ketamine. Doses of psilocybin (0.3 and 1 mg/kg), psilocin, and norpsilocin (0.22 and 0.72 mg/kg) were based on an earlier work (Jefsen et al. 2019), who noted that psilocybin and psilocin can be considered as equipotent at equimolar doses and that due to the different molar masses, psilocin is approximately 1.4 times more potent per milligram than psilocybin. All compounds were given intraperitoneally (IP) 20 min before the test session in the volume of 1 ml/kg.

## Results

### Effects of (R)-ketamine on temporal discrimination

**Fig. 1:**
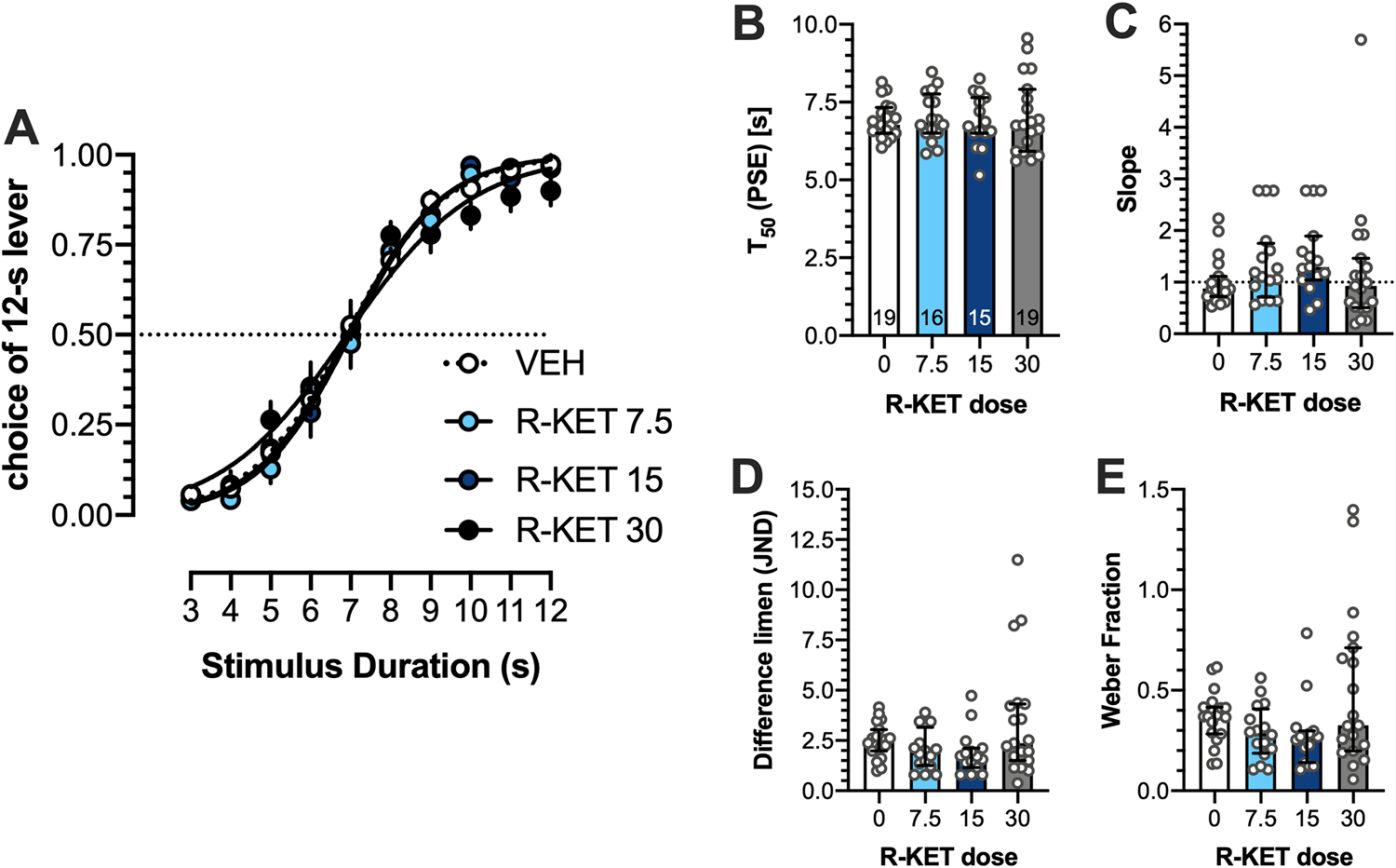
No effects of (*R*)-ketamine on temporal discrimination. (**A**) Mean ± SEM proportion of choices of the 12-s B lever as a function of stimulus duration. (**B**) Mean ± SEM T_50_ (PSE, point of subjective equality). (**C**) Median ± interquartile range of the slope function. (**D**) Median ± interquartile range of the difference limen (JND, just noticeable difference). (**E**) Median ± interquartile range of the Weber fraction. Wherever possible, the number of animals is shown on the bottom of the bar

Two-way ANOVA demonstrated no significant interaction between stimulus duration and (*R*)-ketamine dose: (*F*(27,610) = 0.908; Fig. 1A). There were no effects on T_50_, (*F*(3,65) = 0.1655; Fig. 1B), slope (Kruskal-Wallis 6.903 (4); *P* = 0.0751 Fig. 1C), difference limen (Kruskal-Wallis 6.882 (4); *P* = 0.0757) Fig. 1D), and Weber fraction (Kruskal-Wallis 6.458 (4); *P* = 0.0913 Fig. 1E).

### Effects of (R)-ketamine on response and reward latencies and on omissions, and correct and incorrect responses in the TDT

**Fig. 2:**
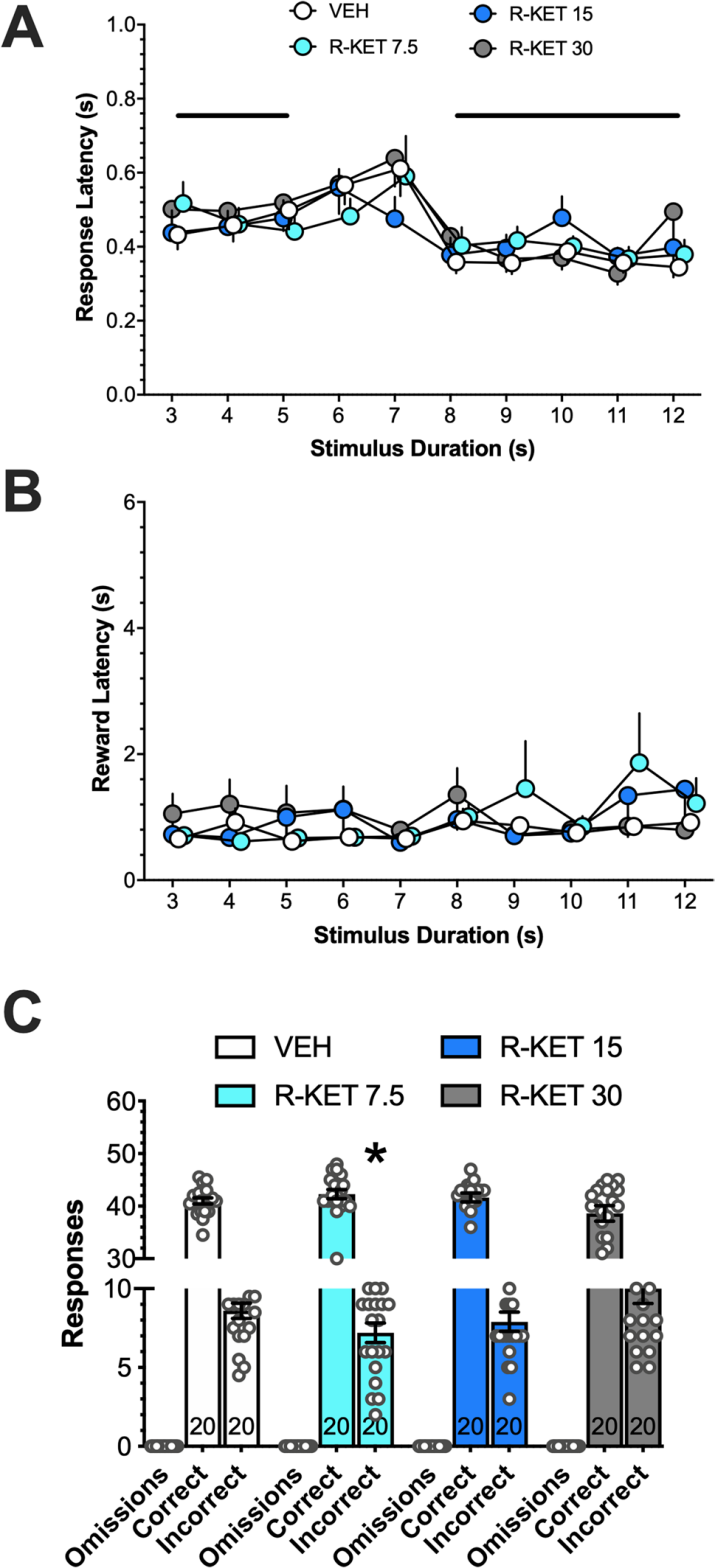
Effects of (*R*)-ketamine on response latency in the temporal discrimination task (**A**). While the dose did not affect response latency, the latencies for stimuli of 3–5 s and 8–12 s were shorter than for the 7-s stimulus (bold lines; *P* < 0.05). (*R*)-Ketamine did not affect reward latency (**B**) but at the dose of 7.5 mg/kg it reduced (*, *P* < 0.05) incorrect responses (**C**). Data are presented as means +, −, or ± SEM. The number of rats tested is shown at the bottom of bars

Two-way ANOVA demonstrated no significant interaction between (*R*)-ketamine dose and stimulus duration for response latencies (*F*(27,753) = 0.571; Fig. 2A). However, response latencies were affected by stimulus duration (*F*(9,753) = 6.777; *P* < 0.001) and post hoc analyses revealed that as compared with the stimulus of 7-s duration, all other stimuli, except 6-s stimulus, resulted in the shorter response latencies (*P* < 0.05, LSD test), suggesting that at stimulus of 7 s, the animals exhibited longer time to respond. Neither (*R*)-ketamine dose, stimulus duration, nor the interaction between these factors affected *reward latencies* (Fig. 2B). Response categories (Fig. 2C) were affected by (*R*)-ketamine dose (*F*(6,114) = 3.657; *P* = 0.02) and as compared with vehicle (8.6 ± 0.48) (*R*)-ketamine at 7.5 mg/kg reduced incorrect responses to 7.2 ± 0.62 (*P* < 0.05, LSD test).

### Effects of (S)-ketamine on temporal discrimination

**Fig. 3:**
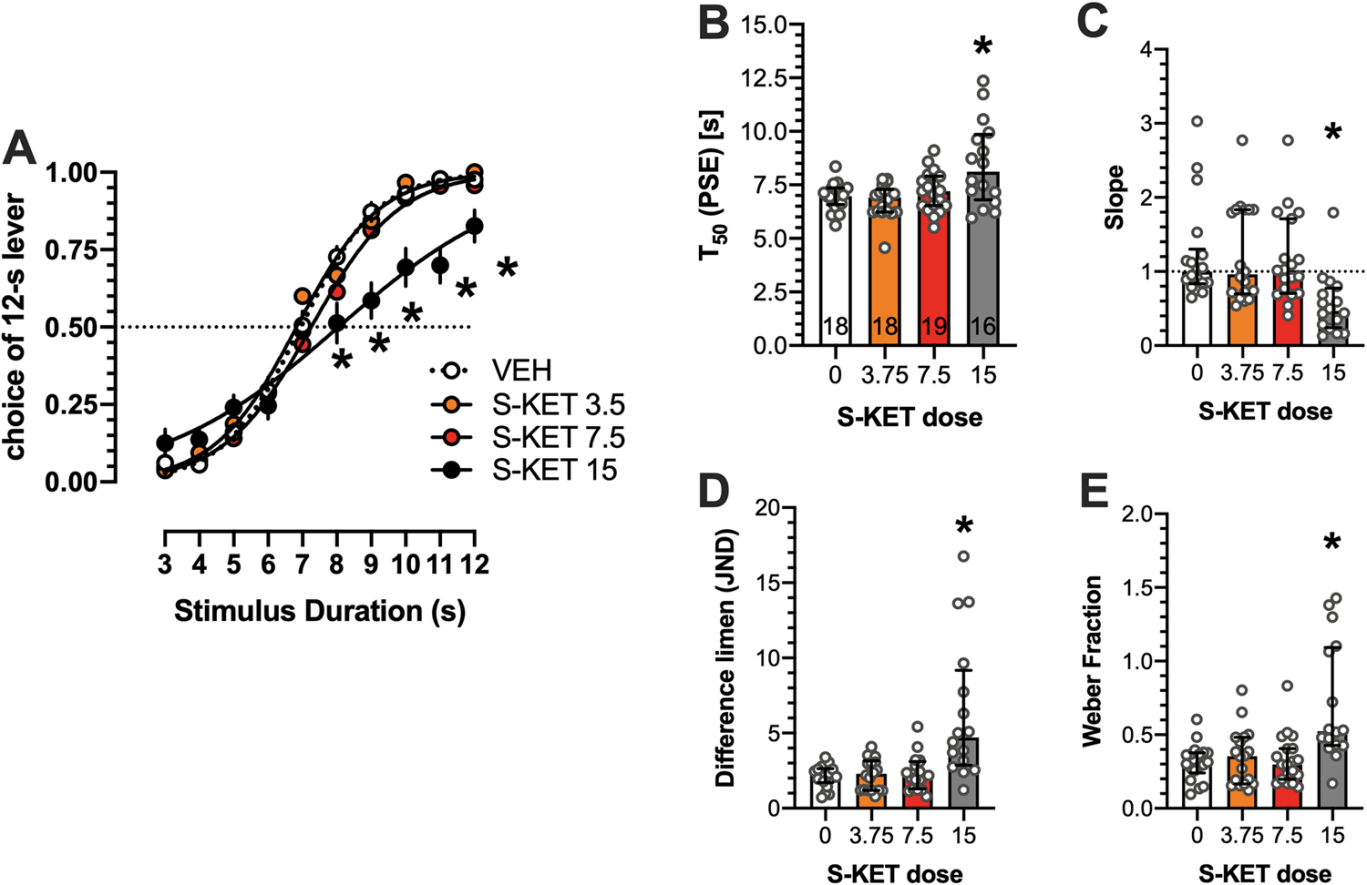
Effects of (*S*)-ketamine on temporal discrimination. (**A**) Mean ± SEM proportion of choices of the 12-s B lever as a function of stimulus duration. (*S*)-Ketamine at the dose of 15 mg/kg shifted the psychometric curve to the right and down, suggesting time underestimation, expressed also as increased mean T_50_ (**B**). The same dose, however, reduced median of the slope function (**C**); increased the median of the difference limen (**D**); as well as the median of the Weber fraction (**E**). *Symbols*: *P < 0.05 versus vehicle. Wherever possible, the number of animals is shown on the bottom of the bar

Two-way ANOVA demonstrated a significant interaction between stimulus duration and ketamine dose (*F*(27,617) = 3.448; *P* < 0.001). At 15 mg/kg, (*S*)-ketamine-treated animals interpreted the stimuli of 8–12-s durations as the “long,” i.e., 12-s anchor stimulus less often (*P* < 0.05; LSD test) than vehicle-treated animals (Fig. 3A).

One-way ANOVA revealed significant effects of (*S*)-ketamine on T_50_, (*F*(3,67) = 7.211; *P* < 0.001); at 15 mg/kg, the compound significantly increased this measure (*P* < 0.05, Dunnett’s multiple comparisons test; Fig. 3B). (*S*)-Ketamine at 15 mg/kg reduced the slope of the psychometric curve (*P* < 0.05; Dunn’s multiple comparisons test following Kruskal-Wallis 19.71 (4); *P* < 0.001 ANOVA; Fig. 3C); it increased difference limen (*P* < 0.05; Dunn’s multiple comparisons test following Kruskal-Wallis 19.59 (4); *P* < 0.001 ANOVA; Fig. 3D) and increased Weber fraction (*P* < 0.05; Dunn’s multiple comparisons test following Kruskal-Wallis 17.57 (4); *P* < 0.001 ANOVA Fig. 3E).

### Effects of (S)-ketamine on response and reward latencies and on omissions, and correct and incorrect responses in the TDT

At 15 mg/kg, the compound increased response latencies and independently on the dose, response latencies for stimuli of 8–12 s were shorter than for the 7-s stimulus. At the dose of 7.5 mg/kg, (*S*)-ketamine increased reward latency. (*S*)-Ketamine at 15 mg/kg reduced correct responses and increased incorrect responses as compared with vehicle (Supplemental Figure 2).

### Effects of psilocybin on temporal discrimination

**Fig. 4:**
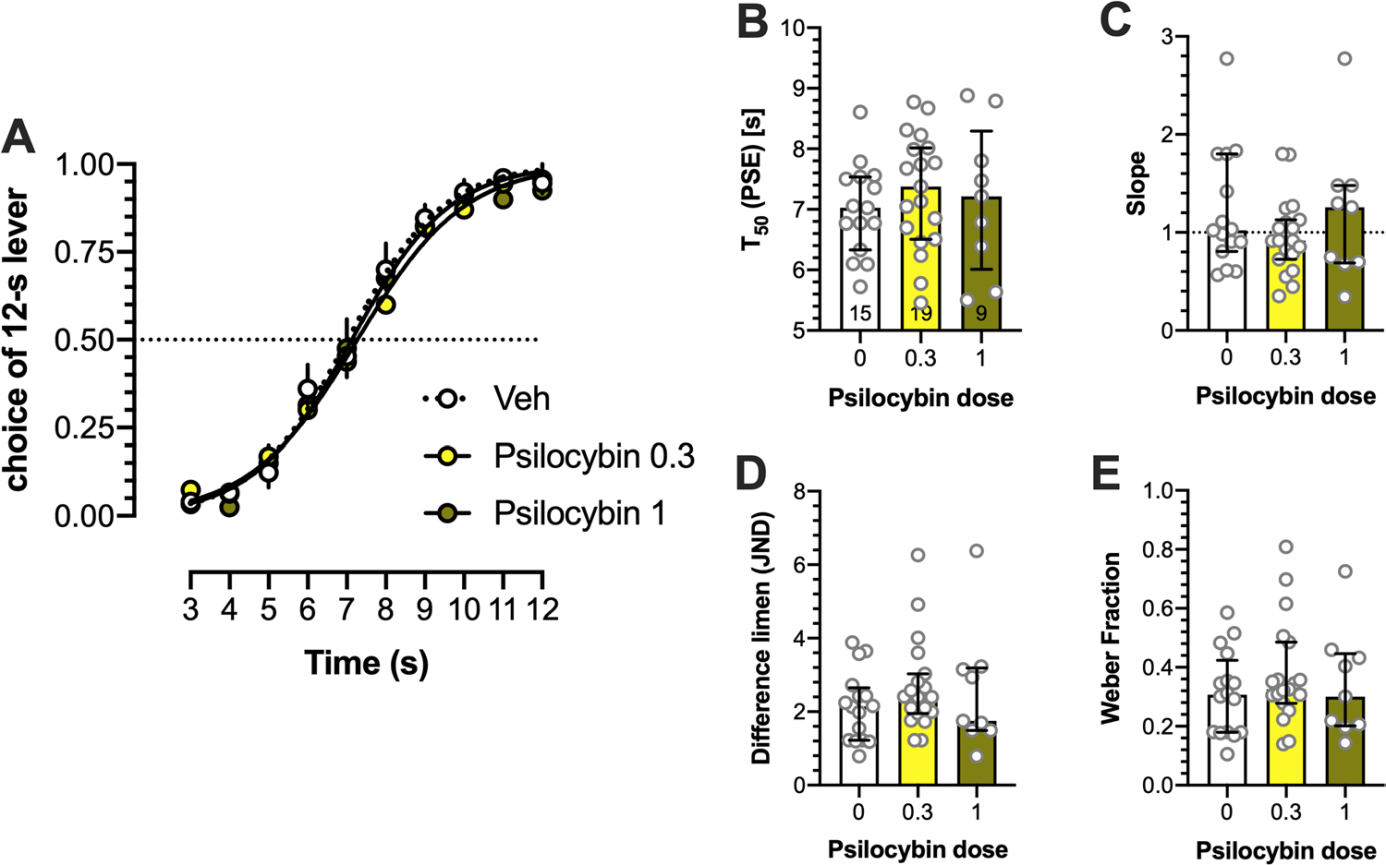
No effects of psilocybin on temporal discrimination. (**A**) The proportion of choices of the 12-s B lever as a function of stimulus duration, (**B**) T_50_, (**C**) slope function, (**D**) difference limen, and (**E**) Weber fraction. Data are shown as means ± SEM or medians ± interquartile range. Wherever possible, the number of animals is shown on the bottom of the bar

Psilocybin affected neither T_50_, slope function, difference limen, nor Weber fraction (Fig. 4). Two-way ANOVA demonstrated no significant interaction between stimulus duration and psilocybin dose: (*F*(18,369) = 0.373; Fig. 4A). There were no effects on T_50_, (*F*(2,40) = 0.418; Fig. 4B), slope (Kruskal-Wallis 1.13 (3); Fig. 4C), difference limen (Kruskal-Wallis 1.684 (3); Fig. 4D), and Weber fraction (Kruskal-Wallis 1.207 (3); Fig. 4E).

At 1 mg/kg, the compound increased response latencies and independently on the dose, response latencies for stimuli of 9–12 s were shorter than for the 7-s stimulus. At the dose of 1 mg/kg, psilocybin increased reward latency. Psilocybin at 1 mg/kg increased omissions and reduced correct responses as compared with vehicle (Supplemental Figure 3).

### Effects of psilocin on temporal discrimination

Psilocin affected neither T_50_, slope function, difference limen, nor Weber fraction (Supplemental Figure 4). At 0.72 mg/kg, the compound increased response latencies and independently on the dose, response latencies for stimuli of 8–9 and 11–12 s were shorter than for the 7-s stimulus. At the dose of 0.72 mg/kg, psilocin increased reward latency. Analyses of response categories revealed that psilocin at 0.72 mg/kg increased omissions and reduced correct responses (Supplemental Figure 5).

### Effects of norpsilocin on temporal discrimination

Norpsilocin affected neither T_50_, slope function, difference limen, nor Weber fraction (Supplemental Figure 6). While the dose of norpsilocin did not affect response latency, the latencies for stimuli of 8–12 s were shorter than for the 7-s stimulus. The compound affected neither reward latency nor response categories (Supplemental Figure 7).

### Effects of (R)- and (S)-ketamine in 5-CSRTT with different stimuli durations

We found higher accuracies for longer (2-s) than for shorter (1-s) stimuli durations but no effect of (*R*)- and (*S*)-ketamine on this measure. Correct responses were decreased by both ketamine enantiomers; (*S*)-ketamine produced stronger effects on this measure, and increasing Sd to 2 s reduced this effect for both isomers although for (*S*)-ketamine at 15 mg/kg this was not the case. Incorrect responses were not affected by ketamine isomers though weakly reduced due to increased stimulus duration. In contrast, response omissions were increased by both isomers, (*S*)-ketamine produced stronger effects on this measure, and increasing Sd to 2 s reduced this effect for both isomers although for (*S*)-ketamine at 15 mg/kg this was not the case (Supplemental Figure 8). Premature responses were unaltered by (*R*)- and (*S*)-ketamine and by stimulus duration, suggesting no effect on impulsivity-like behavior. We found no effect of ketamine enantiomers’ doses and stimulus duration on perseverative responses. In contrast, the high dose of (*S*)-ketamine increased timeout responses (Supplemental Figure 9). The latencies to correct responses were prolonged by the highest doses of (*R*)- and (*S*)-ketamine and in the latter case by increased stimulus duration, suggesting unspecific behavioral effects. The latencies to incorrect responses were also increased by the highest dose of (*S*)-ketamine, but reduced in 2-s Sd conditions. In addition, we found no effects of ketamine enantiomers on reward latency (Supplemental Figure 10).

### Effects of psilocybin and psilocin in 5-CSRTT with different stimuli durations

We found no effects of psilocybin and psilocin on accuracy in 5-CSRTT with different stimulus duration conditions, but decreased correct responses for the highest doses of both compounds. The same doses reduced also incorrect responses, which in case of psilocin (0.22 mg/kg) and its vehicle, were, in addition, reduced in Sd 2-s conditions. The highest doses of psilocybin and psilocin dramatically increased response omissions that were reduced for 0.72 mg/kg of psilocin in Sd 2-s conditions (Supplemental Figure 11). Premature responses were decreased by psilocin (0.72 mg/kg), suggesting a reduction of impulsivity-like behavior in the present experimental conditions. The same dose of psilocin reduced also perseverative responses suggesting reduced compulsive-like behavior. We also found decreased timeout responses due to the high doses of psilocybin and psilocin (Supplemental Figure 12). Latencies to correct responses were prolonged by the highest doses of psilocybin and psilocin; in the case of 0.72 mg/kg of psilocin, they were further increased in Sd 2-s conditions, suggesting unspecific behavioral effects. Latencies to incorrect responses were increased by the highest dose of psilocin in both stimulus duration conditions. We also found increased reward latencies by highest doses of either compounds, suggesting unspecific effects or impaired motivation (Supplemental Figure 13).

### Effects of norpsilocin in 5-CSRTT with standard 1-s stimulus duration conditions

Norpsilocin affected none of the parameters measured in the standard Sd 1-s conditions (Supplemental Figure 14).

### Effects of (R)- and (S)-ketamine on accuracy, omissions, premature, and perseverative responses in 5-CSRTT in variable ITI conditions

#### Accuracy

**Fig. 5:**
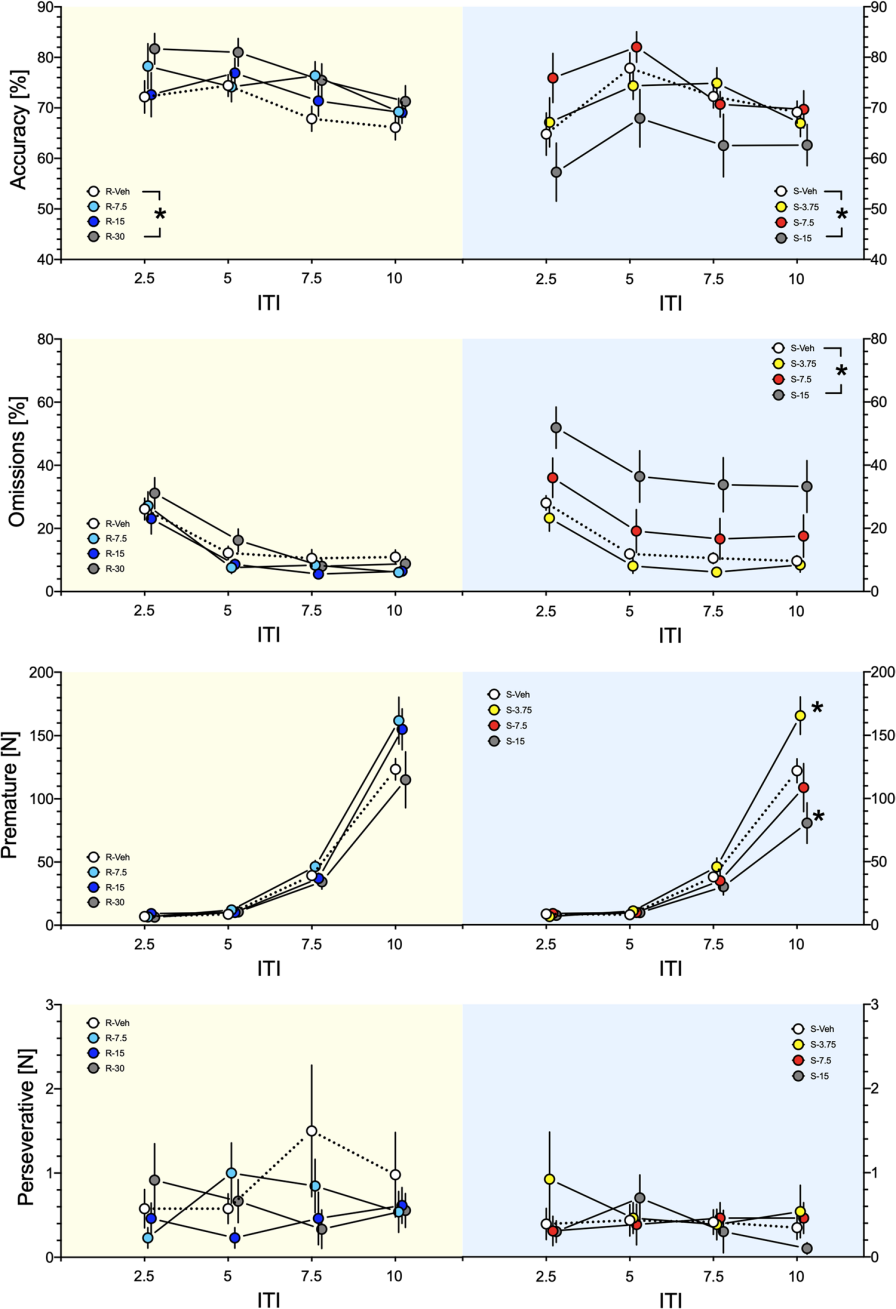
The *top panel* shows that while the highest dose of (*R*)-ketamine increased accuracy in 5-CSRTT, for (*S*)-ketamine, accuracies were decreased (*; *P* < 0.01–0.001, *in legend*), suggesting opposite effects of isomers on this measure of cognitive functions in variable ITI conditions. *Panel close to top* shows no effects of (*R*)-ketamine on response omissions but their increase by the highest dose of (*S*)-ketamine (*; *P* < 0.001, *in legend*), suggesting unspecific effects of this isomer. While premature responses were not affected by (*R*)-ketamine, (*S*)-ketamine in ITI of 10 s increased them at 3.75 mg/kg and decreased them at 15 mg/kg (*; *P* < 0.001; *panel close to bottom*), suggesting increased and decreased effects on impulsivity. Neither compound affected perseverant responses (*bottom panel*). Data represent mean ± SEM. Yellow backgrounds indicate the effects of (*R*)-ketamine; blue backgrounds of (*S*)-ketamine

For (*R*)-ketamine, ANOVA showed no significant interaction between the dose and ITI (*F*(9,292) = 0.488) but a significant dose factor (*F*(3,292) = 8.125; *P* < 0.001); the post hoc test revealed increased accuracy for the dose of 30 mg/kg (*P* < 0.001). For (*S*)-ketamine, ANOVA showed no significant interaction between the dose and ITI (*F*(9,279) = 0.488) but a significant dose factor (*F*(3,279) = 5.931; *P* < 0.001); the post hoc test revealed decreased accuracy for the dose of 15 mg/kg (*P* < 0.01; Fig. 5, top panel).

#### Omissions

For (*R*)-ketamine, ANOVA showed neither significant interaction between the dose and ITI (*F*(9,292) = 0.466) nor the dose factor (*F*(3,292) = 1.990). For (*S*)-ketamine, ANOVA showed no significant interaction between the dose and ITI (*F*(9,284) = 0.025) but a significant dose factor (*F*(3,284) = 23.181; *P* < 0.001); the post hoc test revealed increased omissions for the dose of 15 mg/kg (*P* < 0.001; Fig. 5, panel close to top).

#### Premature responses

For (*R*)-ketamine, ANOVA showed no significant interaction between the dose and ITI (*F*(9,292) = 1.820) but a significant dose factor (*F*(3,292) = 2.800; *P* < 0.05); however, the post hoc test revealed no differences vs. vehicle. For (*S*)-ketamine, ANOVA showed significant interaction between the dose and ITI (*F*(9,284) = 4.292; *P* < 0.001); the post hoc test revealed increased premature responses for the dose of 3.75 mg/kg and decreased premature responses for the dose of 15 mg/kg at ITI of 10 s (*P* < 0.001; Fig. 5, panel close to bottom).

#### Perseverative responses

For (*R*)-ketamine, ANOVA showed neither significant interaction between the dose and ITI (*F*(9,292) = 0.672) nor the dose factor (*F*(3,292) = 0.579). Also for (*S*)-ketamine, neither the interaction (*F*(9,284) = 0.651) nor the dose factor (*F*(3,284)=0.654) were significant; Fig. 5, bottom panel).

### Effects of (R)- and (S)-ketamine on latencies to correct and incorrect responses and on reward latencies in 5-CSRTT in variable ITI conditions

We found no effects of ketamine enantiomers on latencies to correct and incorrect responses. While (*R*)-ketamine did not affect latency to reward, (*S*)-ketamine at the highest dose increased it, suggesting unspecific effects of this isomer (Supplemental Figure 15).

## Discussion

Because depressive patients perceive time flow as slow (i.e., they overestimate time; (Grondin 2020)), we hypothesized that rapid-acting antidepressants could produce an opposite effect, i.e., time underestimation purportedly contributing to their therapeutic action. Only (*S*)-ketamine, and only at the highest dose investigated (15 mg/kg), produced expected time underestimation, but this action was accompanied by numerous unspecific effects. In contrast, (*R*)-ketamine affected neither timing nor produced substantial unspecific effects in TDT and in 5-CSRTT. Psilocybin and psilocin did not affect timing though they produced merely unspecific effects (Table 3). In contrast, norpsilocin did not exert measurable effects in the TDT and in 5-CSRTT. The lack of effects of psilocybin-like compounds in TDT is unlikely due to the purportedly long-lasting effects of high doses of ketamine enantiomers, as they were tested at the very end of the study.

**Table 3:**
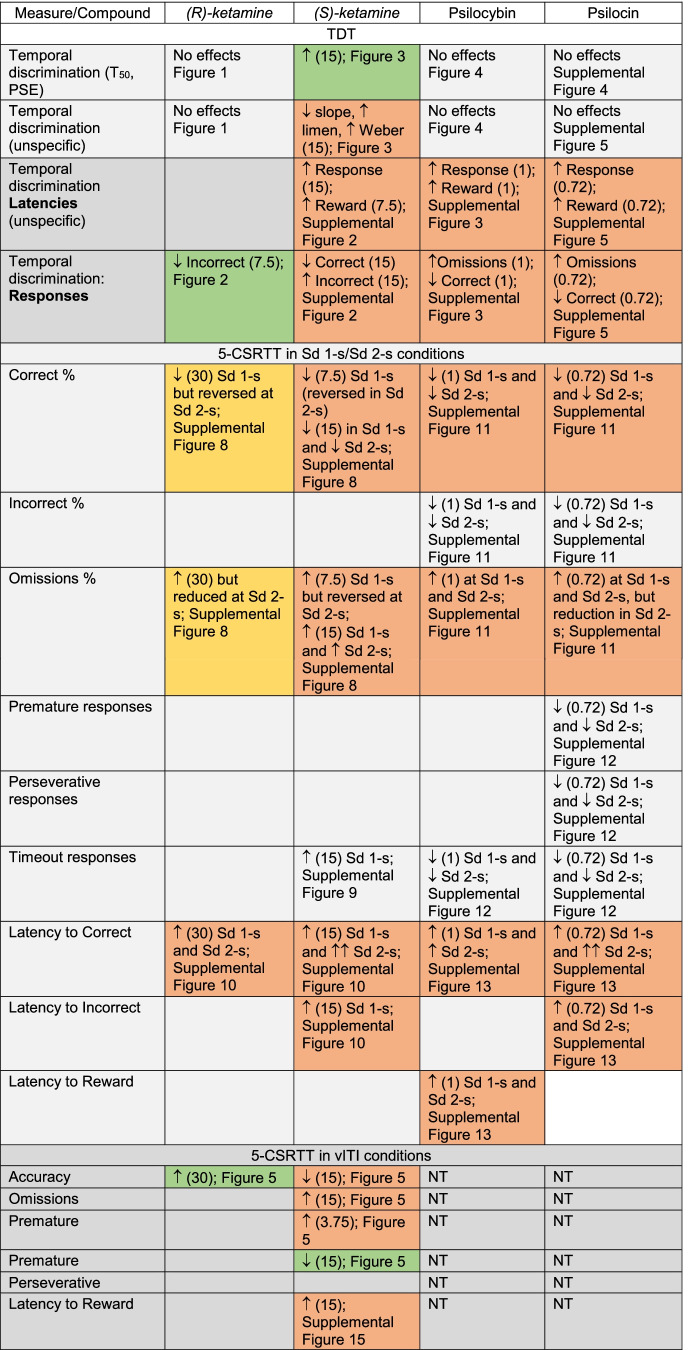
Summary of the effects of (*R*)- and (*S*)-ketamine, psilocybin, and psilocin on time estimation in discrete-trial temporal discrimination task (TDT) and cognition in the 5-CSRTT in the standard conditions of stimulus duration (Sd) of 1 s, easier conditions with Sd of 2 s, as well as in variable inter-trial interval (vITI) variant promoting impulsivity. Actions of norpsilocin are not shown due to the lack of effects. Doses (mg/kg) are shown in parentheses. The green background may suggest possible therapeutic effects (increased timing and lower premature responses for 15 mg/kg of (*S*)-ketamine as well as decreased incorrect responses and increased accuracy for (*R*)-ketamine), the yellow background may suggest that the purportedly unspecific effects of (*R*)-ketamine on correct responses and response omissions are alleviated in the “easier”, 2-s variant of the task, and the red background suggests numerous unspecific effects accompanying or not accompanying the desired effect

Assuming that (*S*)-ketamine, (*R*)-ketamine, and psilocybin produce similar rapid antidepressant effects in clinical settings, it is reasonable to conclude that the hypothesized effects on timing are unlikely to explain their therapeutic action. This is because while (*S*)-ketamine produced time underestimation, neither (*R*)-ketamine, psilocybin, nor psilocin demonstrated similar effects. Alternatively, one can hypothesize that time underestimation produced by (*S*)-ketamine may have unique therapeutic effects. Yet another possibility could be that the robust rapid antidepressant effects are observed clinically only for the (*S*)-ketamine, though this could be shown only in a clinical trial comparing directly both ketamine isomers and psilocybin. Overall, since (*R*)-ketamine in the temporal discrimination task produced no measurable effects on timing but is clinically active (Leal et al. 2020), the question remains whether the psychotomimetic-like effects are at all necessary for the antidepressant actions.

Data collected on vehicle control days in TDT fit well with the existing literature on temporal discrimination task, in which T_50_ usually lies near the geometric mean of the anchor durations (Meck 1997). It is also known that the response latency is longer at the geometric mean of the two anchor signal durations (Maricq et al. 1981; Meck 1983), suggesting that rats are most uncertain about how to classify signal durations in this region (Meck 1997) and/or that the middle stimulus duration produces a conflict (Maricq and Church 1983).

The psychometric curve was right-shifted by (*S*)*-*ketamine (15 mg/kg). This is in some contrast to Cheng et al. (2006) report who found that racemic (*R,S*)-ketamine at a single dose of 15 mg/kg did not affect timing. Assuming that (*R,S*)-ketamine contains 50% of the (*S*)-isomer that displays twice higher affinity for NMDA receptors (Zukin et al. 1984), it is likely that the dose of racemate used by Cheng et al. (2006) was insufficient to affect timing.

(*S*)-Ketamine affected time perception also in 5-CSRTT. While 5-CSRTT has not been specifically designed to examine timing, present experiment with (*S*)-ketamine may shed some interesting light on this issue. In this task, premature responses occur inappropriately, before the visual targets have occurred and presumably during the period, in which the rats are anticipating their occurrence (Evenden 1999; Robbins 2002). In the vITI variant of the task, at long (10 s) ITI, control animals display high number of premature responses because they were trained to wait for the standard 5-s ITI before one of the LEDs begin signaling. However, if for an animal treated with (*S*)-ketamine the time passes subjectively more quickly, an ITI of 10 s would be perceived as a shorter ITI (e.g., of 5 s); thus, the animal would demonstrate less of premature responses. This was exactly what rats treated with 15 mg/kg of (*S*)-ketamine have displayed (Fig. **5**, panel close to bottom). Overall, present findings corroborate the premise that premature responses in 5-CSRTT are related to timing as reported in the case of infrequent no-light trials insertion (Cope et al. 2016).

The leftward shift of the psychometric functions in the TDT (decrease in T_50_) reflects time overestimation. In the case of a low level of behavioral activity characteristic for boredom (Nichelli 1996), the tract of time empty of experiences seems *long in passing* (Wearden 2016) causing the subject to *classify stimuli as being* “*longer*” than normal (Meck 1997). This type of distorted time estimation is reported in depressed patients (Caceda et al. 2020; Grondin 2020). As pointed by Northoff et al. (2018), both phenomenological and psychological investigations show that depressed patients perceive time as extremely slow and retarded relative to control subjects (for a recent meta-analysis, see (Thönes and Oberfeld 2015)). In contrast, (*S*)-ketamine-induced rightward shift of the psychometric functions (increase of T_50_) is interpreted as time underestimation (Maricq et al. 1981). This is observed also in the human time bisection task, in which positive affective states with high approach motivation made time appearing to *pass more quickly*, causing assessments of elapsed time to be *shorter* (Gable and Poole 2012).

However, time underestimation produced by (*S*)-ketamine was confounded by severe disruption of task performance. These deficits appear functionally similar to the effects of physical distractors (like the flashing light in the seminal Amitai and Markou (2011) experiment), which produced comprehensive impairment, including disrupted attention and increased premature and timeout responses. These deficits resemble also effects of racemic ketamine (Benn and Robinson 2014; Gastambide et al. 2013; Higgins et al. 2021; Nemeth et al. 2010; Nikiforuk and Popik 2014; Oliver et al. 2009) and of (*S*)-ketamine (Smith et al. 2011) in the earlier studies.

The effects of other psychedelic-like compounds on time estimation also appear to be crucially associated with unspecific cognitive deficits. For instance, the hallucinogenic 5-HT_2A/2C_ receptor agonist DOI increased T_50_ in temporal discrimination, but it also produced disruptive effects (Asgari et al. 2006), leading the authors to conclude that the deleterious effect on discriminative accuracy could be due to a breakdown of stimulus control. Thus, as in the earlier reports with DOI (Asgari et al. 2006), scopolamine (Berz et al. 1992), and other drugs (Asgari et al. 2006; Maricq et al. 1981), it is likely that (*S*)*-*ketamine affected neural mechanisms of timing but also broke down the stimulus control. Nonetheless, it is disputable whether the inability of an animal to perform the operant task precludes valid conclusions. Instead, disrupted behavior could reflect the reliable pharmacological action, suggestive of a psychotomimetic effect.

Overall, one can hypothesize that underestimated time as in case of (*S*)-ketamine may be associated with the therapeutic effects in depressed patients who perceive time and its speed as extremely slow. The present study suggests, however, that these effects are at the cost of severe unspecific distortions.

In contrast to the (*S*)-isomer, (*R*)*-*ketamine reduced incorrect responses and increased accuracy in the vITI variant of 5-CSRTT. These unexpected pro-cognitive effects warrant further studies that are now undertaken in our laboratory.

## Supplementary Information

Below is the link to the electronic supplementary material.

Supplementary file1 (DOC 3.43 MB)
