# Supplementary material for: Effects of ketamine optical isomers, psilocybin, psilocin and norpsilocin on time estimation and cognition in rats
Source: Psychopharmacology (Berl). 2022 Mar 2;239(6):1689–703. doi: 10.1007/s00213-021-06020-5 (PMC9166826; doi:10.1007/s00213-021-06020-5)
Supplement: Supplementary file 1 — Supplementary file1 (DOC 3.43 MB) [file 213_2021_6020_MOESM1_ESM.docx]

Effects of ketamine optical isomers, psilocybin, psilocin and norpsilocin on time estimation and cognition in rats

Piotr Popik, Adam Hogendorf, Ryszard Bugno, Shaun Yon-Seng Khoo, Pawel Zajdel, Natalia Malikowska-Racia, Agnieszka Nikiforuk, Joanna Golebiowska

## Supplemental Information


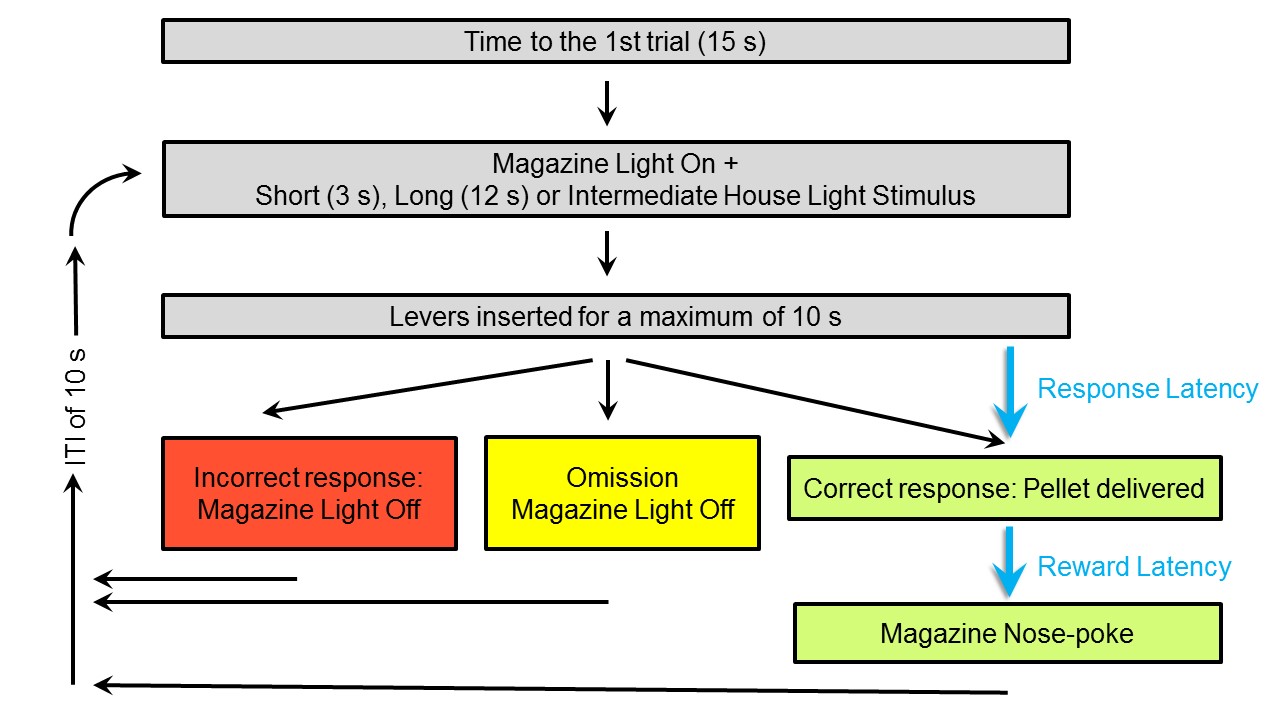


Supplemental Figure 1. The scheme of training and testing sessions in the temporal discrimination task

# Results

## Effects of (*S*)-ketamine on response and reward latencies and on omissions, correct and incorrect responses in the temporal discrimination task


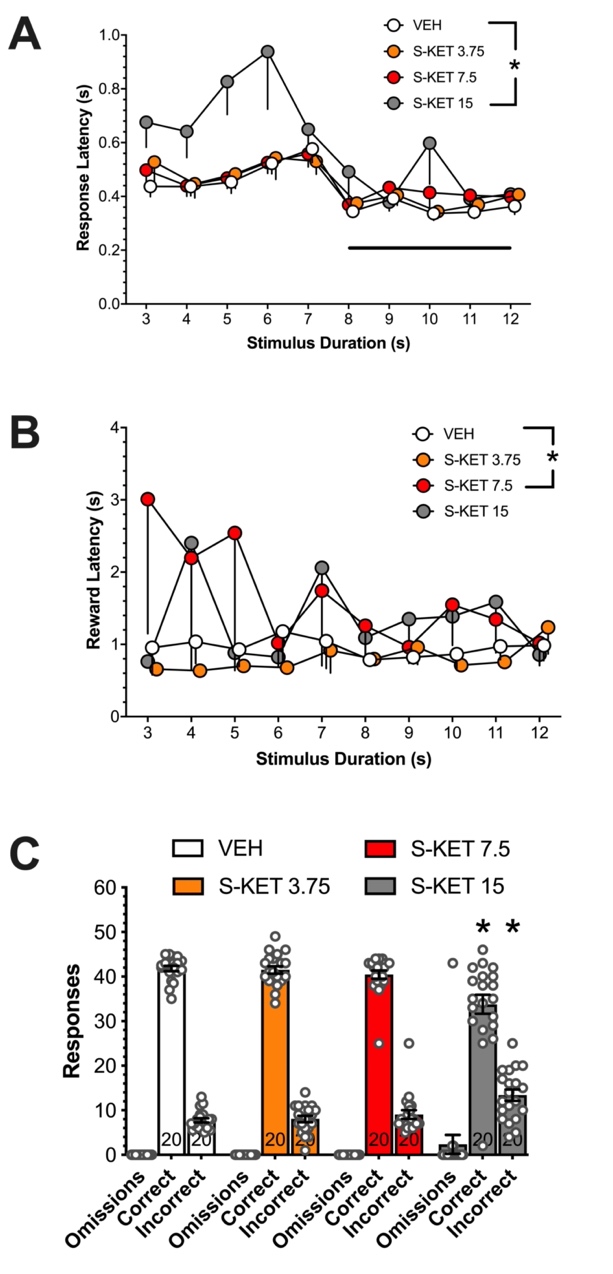


Supplemental Figure 2. (S)-ketamine affects response and reward latencies as well as response categories in the temporal discrimination task. (**A**) At 15 mg/kg the compound increased response latencies (* P<0.05, **in Legend**) and independently on the dose, response latencies for stimuli of 8-12 s were shorter than for the 7-s stimulus (P<0.05, **bold line**). (**B**) At the dose of 7.5 mg/kg (S)-ketamine increased reward latency (*P<0.05 **in Legend**). Analyses of response categories (**C**) revealed that (S)-ketamine at 15 mg/kg reduced correct responses and increased incorrect responses as compared with vehicle (*; P<0.05). Data are presented as means +, - or ± SEM. The number of rats tested is shown at the bottom of bars.

Two-way ANOVA demonstrated no significant interaction between (*S*)-ketamine dose and stimulus duration for response latencies (F(27,748)=1.244, Supplemental Figure 2**A**). However, the dose factor affected response latencies (F(3,748)=14.751; P<0.001) and post-hoc analyses revealed that as compared with vehicle treatment, (*S*)-ketamine at 15 mg/kg increased this measure (P<0.05, LSD test), suggesting unspecific effects. Like the in case of the (*R)*-isomer, stimulus duration affected response latencies (F(9,748)=7.703; P<0.001) and post-hoc analyses revealed that as compared with the stimulus of 7-s duration, stimuli of durations of 8-12 s resulted in shorter response latencies (P<0.05, LSD test).

Supplemental Figure 2**B** shows that (*S*)-ketamine dose affected reward latencies (F(3,748)= 3.849; P<0.01) and that the dose of 7.5 mg/kg significantly increased this measure (P<0.05, LSD test).

Two way ANOVA for response categories (Supplemental Figure 2**C**) revealed a significant interaction between (*S*)-ketamine dose and responses category (F(6,114)=6.338; P<0.001) and post-hoc analyses showed that as compared with vehicle, (*S*)-ketamine at 15 mg/kg reduced correct responses and increased incorrect responses (P<0.05, LSD test).

## Effects of psilocybin on response and reward latencies and on omissions, correct and incorrect responses in the temporal discrimination task


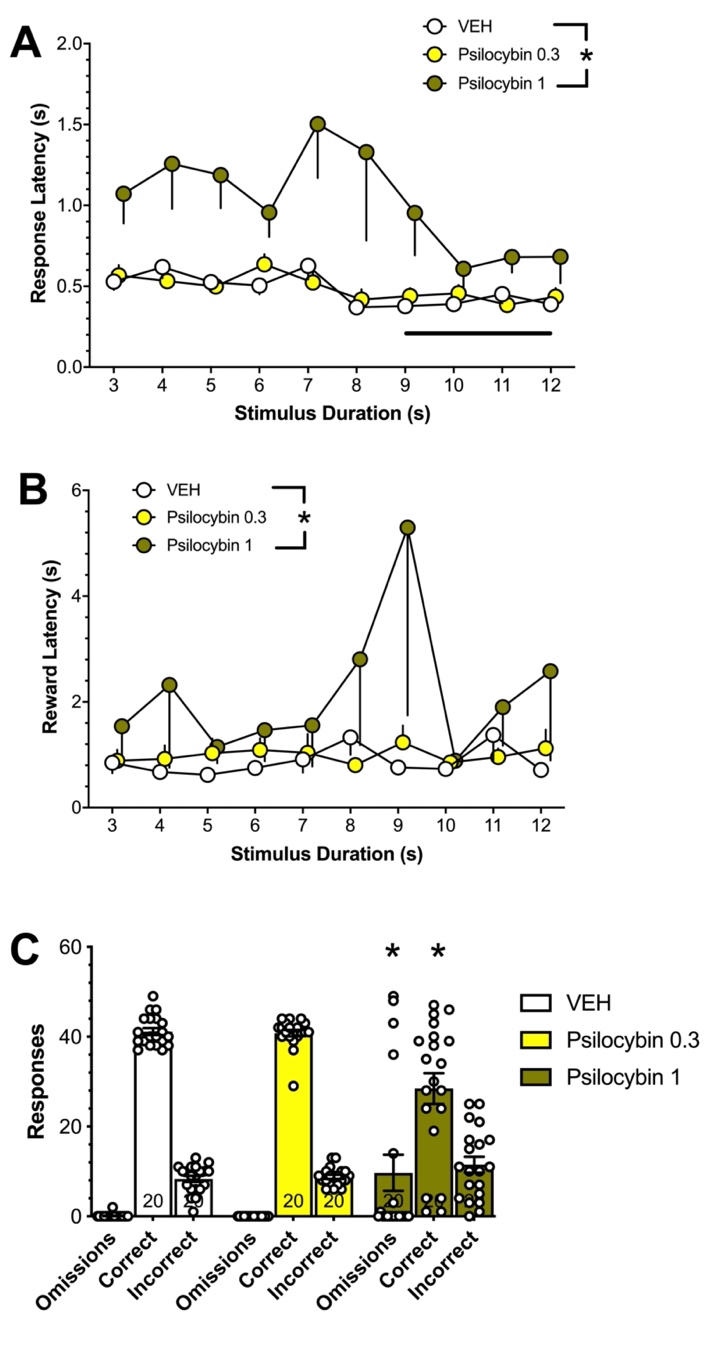


Supplemental Figure 3. Psilocybin affects response and reward latencies as well as response categories in the temporal discrimination task. (**A**): At 1 mg/kg the compound increased response latencies (* P<0.001, **in Legend**) and independently on the dose, response latencies for stimuli of 9-12 s were shorter than for the 7-s stimulus (P<0.05, **bold line**). (**B**) At the dose of 1 mg/kg psilocybin increased reward latency (*P<0.001 **in Legend**). Analyses of response categories (**C**) revealed that psilocybin at 1 mg/kg increased omissions and reduced correct responses as compared with vehicle (*; P<0.05). Data are presented as means +, - or ± SEM. The number of rats tested is shown at the bottom of bars.

Two-way ANOVA demonstrated no significant interaction between psilocybin dose and stimulus duration for response latencies (F(18,532)= 1.162; Supplemental Figure 3**A**), however, the dose factor was significant (F(2,532)=44.959; P<0.001) and LSD post-hoc test demonstrated longer response latencies for psilocybin at 1 mg/kg as compared with vehicle (P<0.001), suggesting unspecific effects exerted by this dose. In addition, response latencies were affected by stimulus duration (F(9,532)= 2.711; P<0.01) and post-hoc analyses revealed that as compared with the stimulus of 7-s, stimuli of 9-12s resulted in the shorter response latencies (P<0.05, LSD test).

Stimulus duration did not affect reward latencies, the interaction between stimulus duration x dose was also no significant (Supplemental Figure 3**B**), however reward latencies were affected by psilocybin dose (F(2,532)=7.459; P=0.001) and the LSD post-hoc test demonstrated longer reward latencies for psilocybin at 1 mg/kg as compared with vehicle (P<0.001), again suggesting unspecific effects exerted by this dose in the TD task.

Psilocybin dose affected response categories (Supplemental Figure 3**C**) as the interaction between these measures was significant (F(4,76)=7.780; P<0.001) and as compared with vehicle, psilocybin at 1 mg/kg increased omissions and reduced correct responses (P<0.05, LSD test).

## Effects of psilocin on temporal discrimination


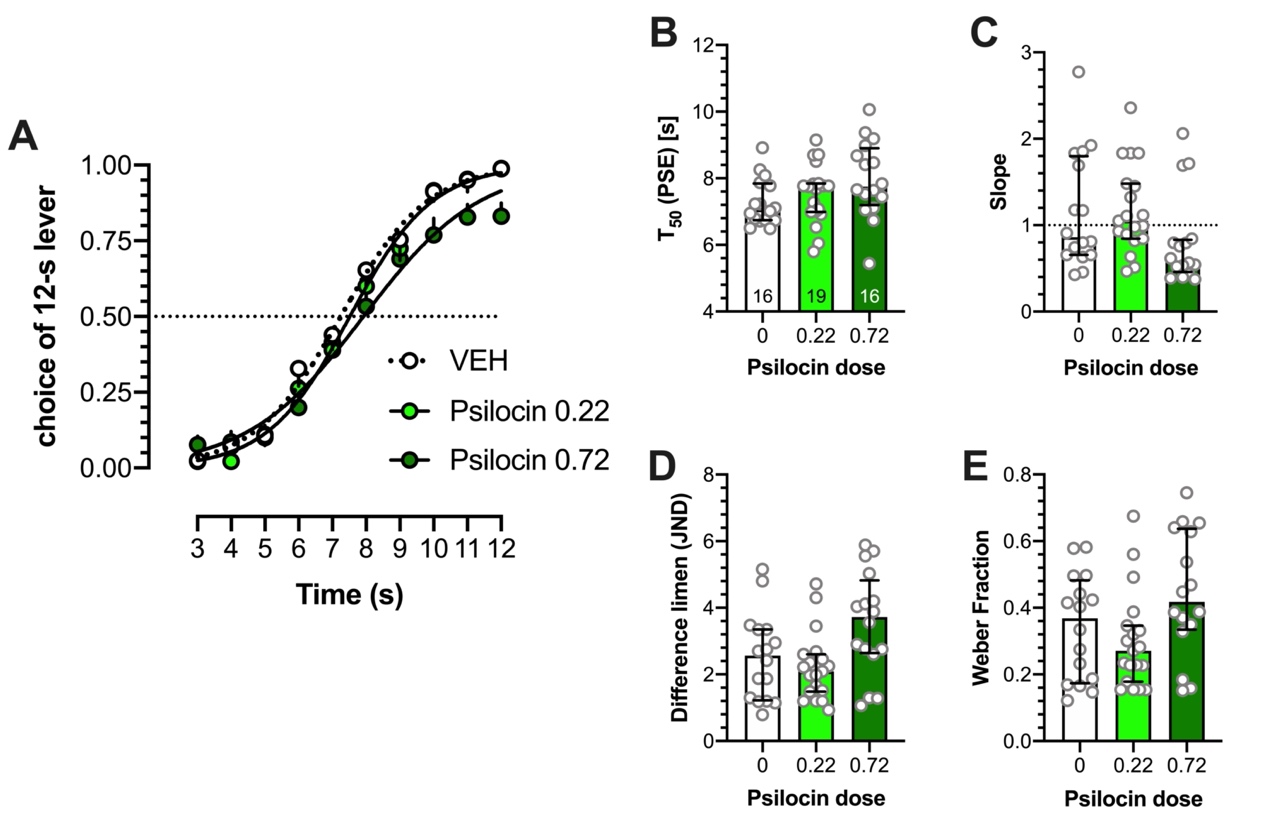


Supplemental Figure 4. No effects of psilocin on temporal discrimination. (**A**) The proportion of choices of the 12-s B lever as a function of stimulus duration, (**B**) T_50_, (**C)** slope function, (**D**) difference limen, and (**E)** Weber fraction. Data are shown as means ± SEM or medians + interquartile range. Wherever possible, the number of animals is shown on the bottom of the bar.

Two way ANOVA demonstrated no significant interaction between stimulus duration and psilocin dose: (F(18,435)=1.133; Supplemental Figure 4**A**). There were no effects on T_50_, (F(2,48) = 2.311; Supplemental Figure 4**B**), but significant effects on slope (Kruskal-Wallis 7.606 (3); P<0.05; Supplemental Figure 4**C**), difference limen (F(2,48=4.792; P<0.05; Supplemental Figure 4**D**), and Weber fraction (F(2,48)=3.505; P<0.05; Supplemental Figure 4**E**). However, in these measures, post-hoc analyses did not indicate significant differences as compared with vehicle’s effects.

## Effects of psilocin on response and reward latencies and on omissions, correct and incorrect responses in the temporal discrimination task


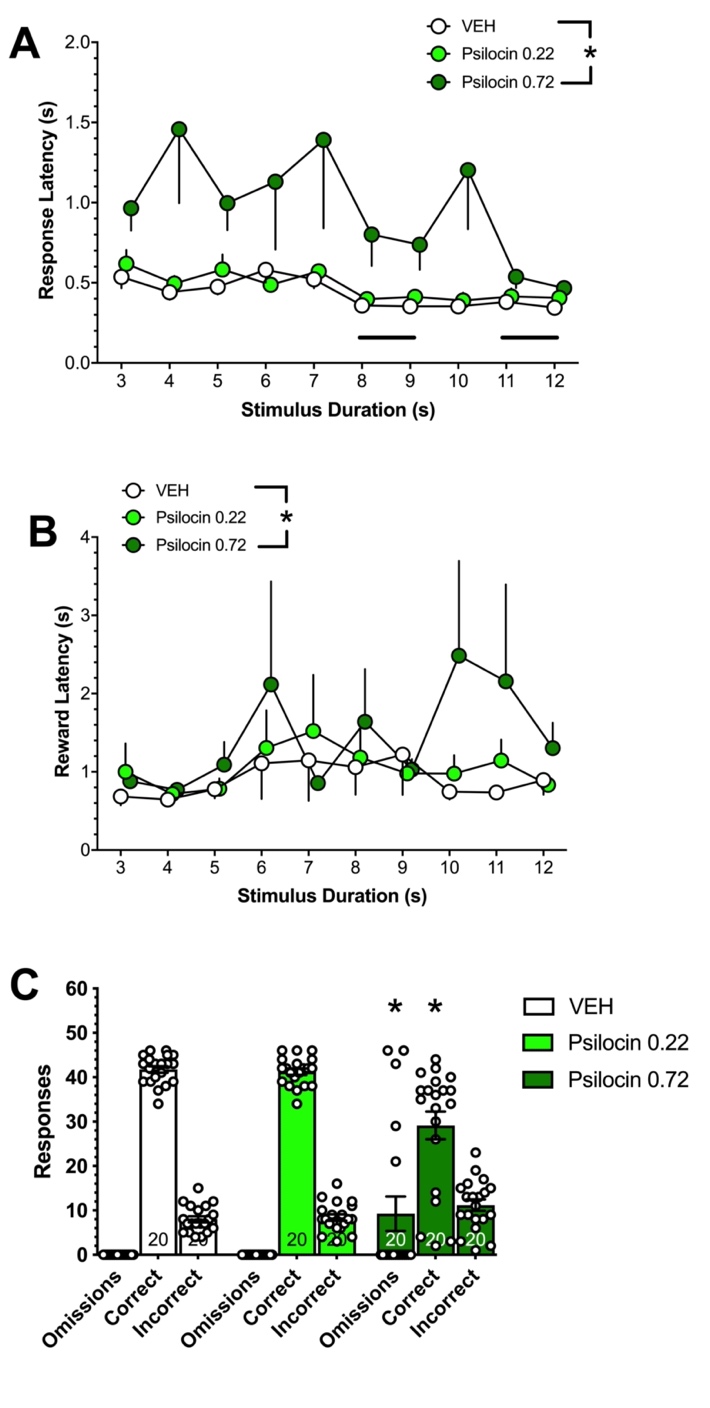


Supplemental Figure 5. Psilocin affects response and reward latencies as well as response categories in the temporal discrimination task. (**A**): At 0.72 mg/kg the compound increased response latencies (*P<0.001, **in Legend**) and independently on the dose, response latencies for stimuli of 8-9 and 11-12-s were shorter than for the 7-s stimulus (P<0.05, **bold lines**). (**B**) At the dose of 0.72 mg/kg psilocin increased reward latency (*P<0.05 **in Legend**). Analyses of response categories (**C**) revealed that psilocin at 0.72 mg/kg increased omissions (*, P<0.05) and reduced correct responses (*, P<0.05). Data are presented as means +, - or ± SEM. The number of rats tested is shown at the bottom of bars.

Two-way ANOVA demonstrated no significant interaction between psilocin dose and stimulus duration for response latencies (F(18,541)= 1.007; Supplemental Figure 5**A**), however, the dose factor was significant (F(2,541)=28.532; P<0.001) and LSD post-hoc test demonstrated longer response latencies for psilocin at 0.72 mg/kg as compared with vehicle (P<0.001), suggesting unspecific effects exerted by this dose. In addition, response latencies were affected by stimulus duration (F(9,541)= 2.262; P<0.05) and LSD post-hoc analyses revealed that as compared with the stimulus of 7-s, stimuli of 8-9-s and 11-12-s resulted in shorter response latencies (P<0.05).

Stimulus duration did not affect reward latencies, the interaction between stimulus duration x dose was also no significant (Supplemental Figure 5**B**), however reward latencies were affected by psilocin dose (F(2,539)=3.118; P<0.05) and the LSD post-hoc test demonstrated longer reward latencies for psilocin at 0.72 mg/kg as compared with vehicle (P<0.05), again suggesting unspecific effects exerted by this dose in the TD task.

Psilocin dose affected response categories (Supplemental Figure 5**C**) as the interaction between these measures was significant (F(4,76)=8.924; P<0.001) and as compared with vehicle, psilocin at 0.72 mg/kg increased omissions and reduced correct responses (P<0.05, LSD test).

## Effects of norpsilocin on temporal discrimination


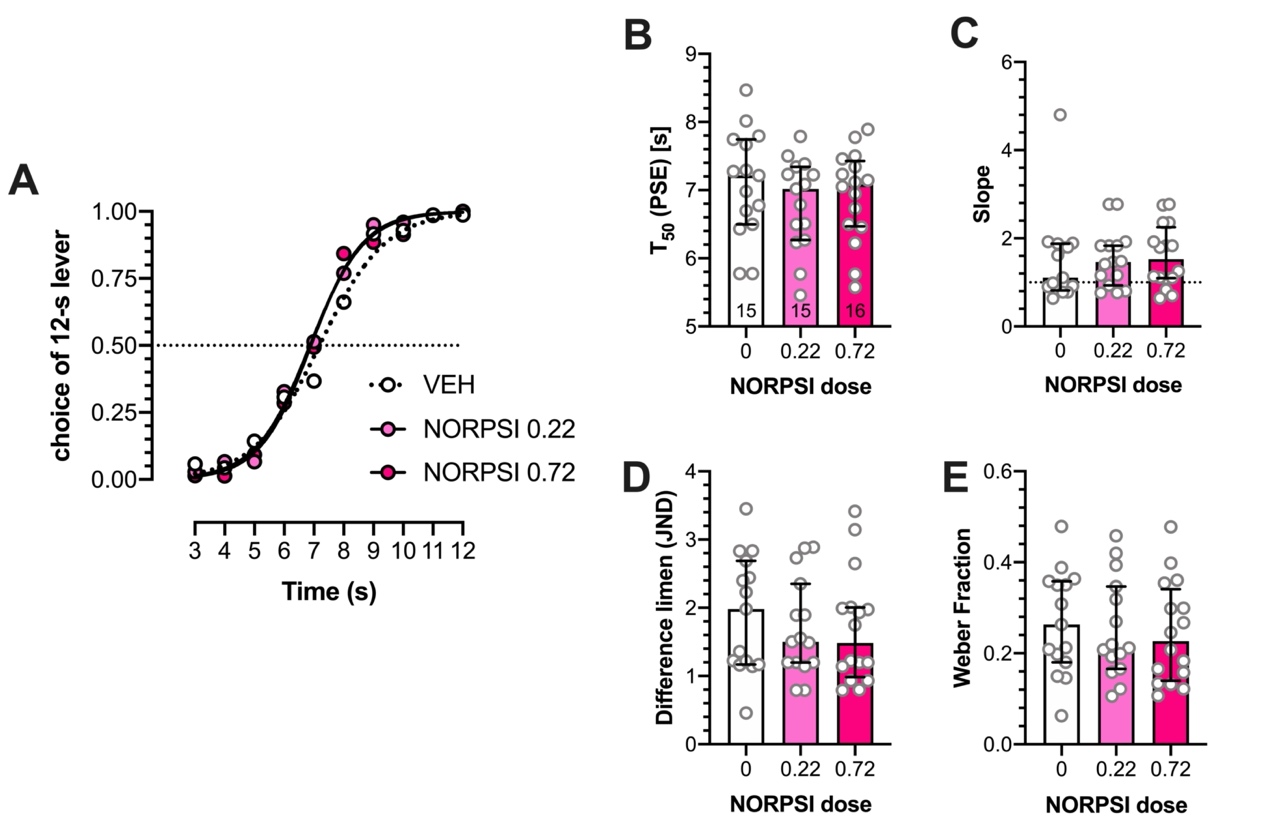


Supplemental Figure 6. No effects of norpsilocin on temporal discrimination. (**A**) The proportion of choices of the 12-s B lever as a function of stimulus duration, (**B**) T_50_, (**C)** slope function, (**D**) difference limen, and (**E)** Weber fraction. Data are shown as means ± SEM or medians + interquartile range. Wherever possible, the number of animals is shown on the bottom of the bar.

Two way ANOVA demonstrated no significant interaction between stimulus duration and norpsilocin dose: (F(18,380)=1.130; Supplemental Figure 6**A**). There were no effects on T_50_, (F(2,43) = 0.62; Supplemental Figure 6**B**), slope (Kruskal-Wallis 0.88 (3); Supplemental Figure 6**C**), difference limen (F(2,43)=0.34; Supplemental Figure 6**D**), and Weber fraction (F(2,43)=0.17; Supplemental Figure 6**E**).

## Effects of norpsilocin on response and reward latencies and on omissions, correct and incorrect responses in the temporal discrimination task


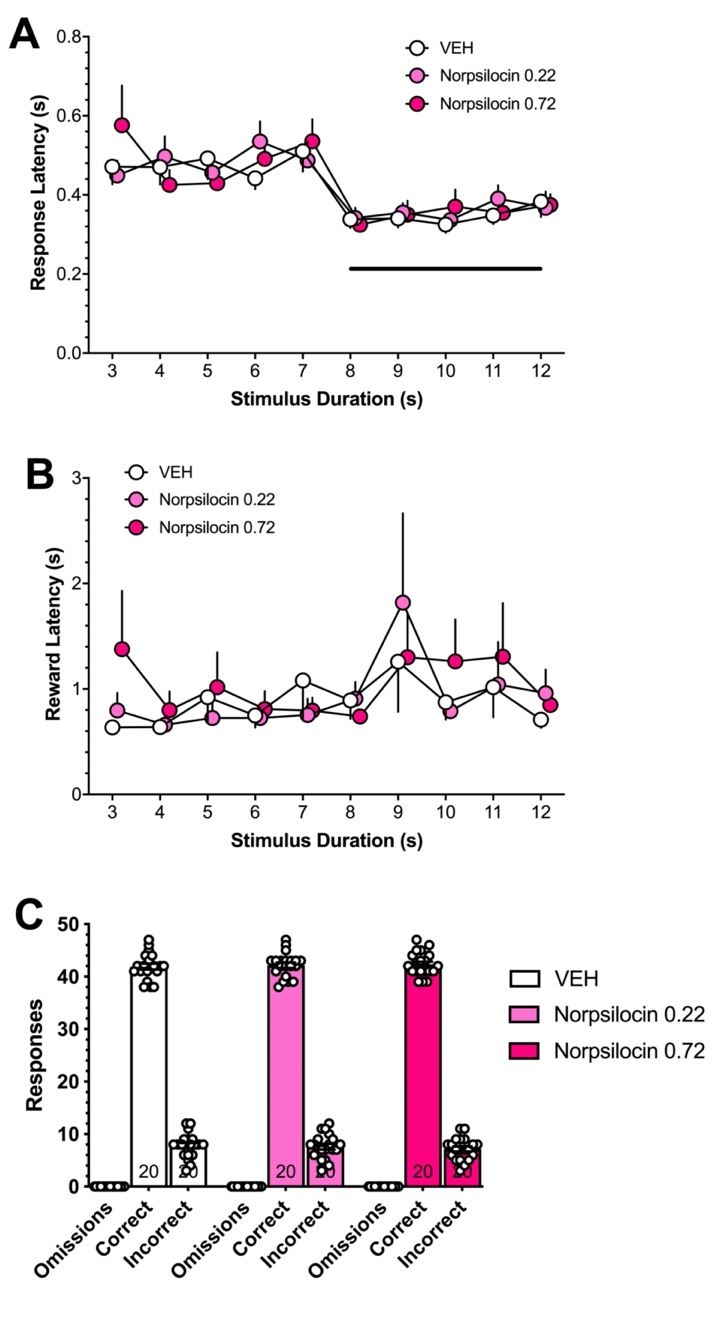


Supplemental Figure 7. No effects of norpsilocin on response latency in the temporal discrimination task (**A**). While the dose did not affect response latency, the latencies for stimuli of 8-12 s were shorter than for the 7-s stimulus (P<0.05, **bold line**). Norpsilocin affected neither reward latency (**B**) nor response categories (**C**). Data are presented as means +, - or ± SEM. The number of rats tested is shown at the bottom of bars.

Two-way ANOVA demonstrated no significant interaction between norpsilocin dose and stimulus duration for response latencies (F(18,560)= 0.715; Supplemental Figure 7**A**), the dose factor was also no significant. Significant stimulus duration factor (F(9,560)=9,172; P<0.001) allowed for post-hoc analyses, which revealed that as compared with the stimulus of 7-s, stimuli of 8-12-s resulted in shorter response latencies (P<0.05, LSD test).

Neither norpsilocin dose, stimulus duration nor their interaction affected reward latencies in the TD task (Supplemental Figure 7**B**), suggesting lack of unspecific effects of this compound.

Norpsilocin dose did not affect response categories (Supplemental Figure 7**C**) as the interaction between these measures was significant (F(4,76)=0.329).

## Effects of (*R*)- and (*S*)-ketamine on accuracy and correct and incorrect responses and on omissions in 5-CSRTT with different stimuli durations


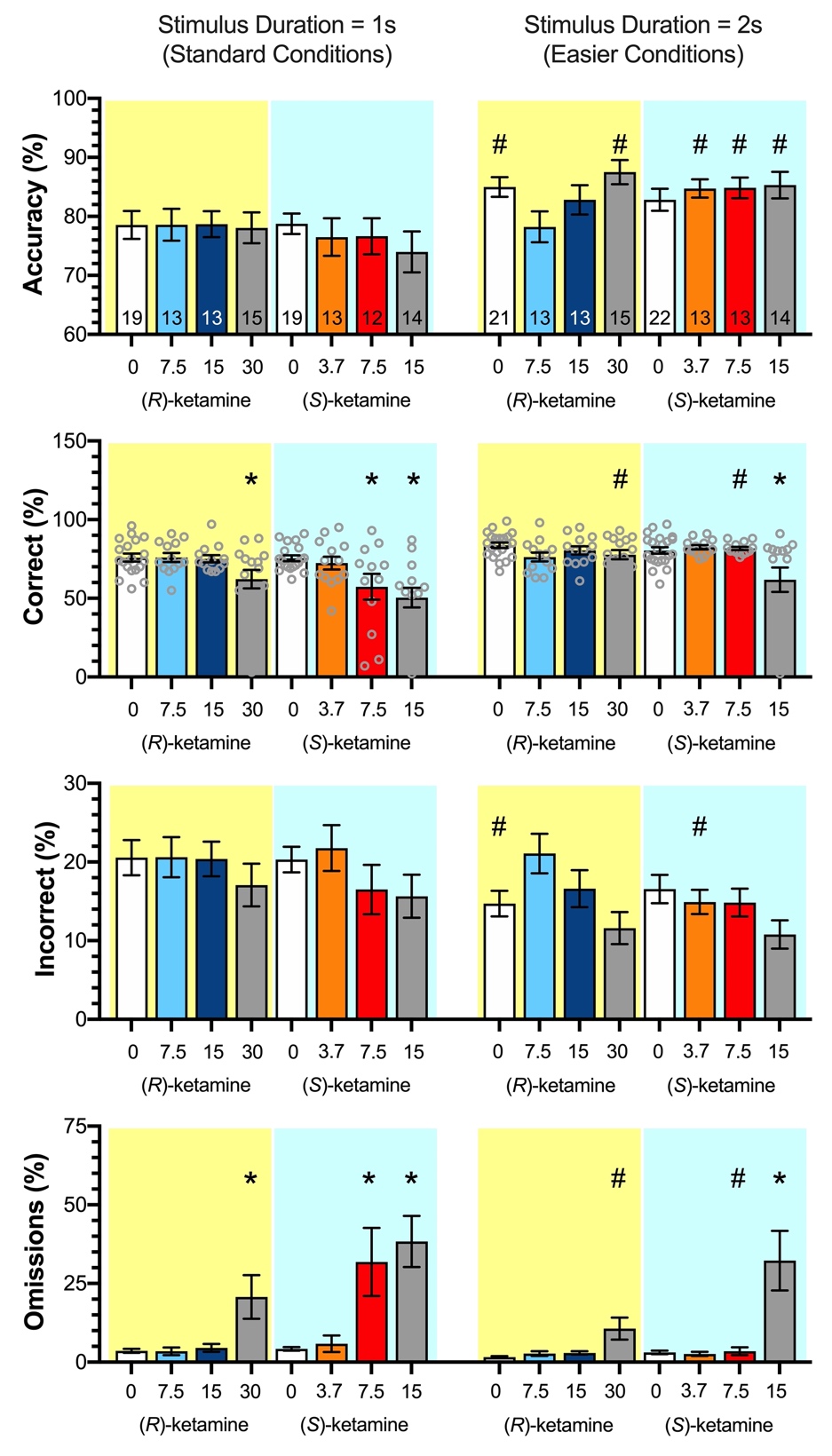


Supplemental Figure 8. The **top panel** shows higher accuracies for longer (2s) than for shorter (1s) stimuli durations and no effect of (R)- and (S)-ketamine on this measure in a given experimental condition. **Panel close to top** shows correct responses, that both isomers decreased them, that (S)-ketamine produced stronger effects on this measure, and that increasing Sd to 2 s reduced this effect for both isomers although for (S)-ketamine at 15 mg/kg this was not the case. **Panel close to bottom** shows incorrect responses that were not affected by ketamine isomers though weakly reduced due to increased stimulus duration. The **bottom panel** shows response omissions apparently increased by both isomers, that (S)-ketamine produced stronger effects on this measure, and that increasing Sd to 2 s reduced this effect for both isomers although for (S)-ketamine at 15 mg/kg this was not the case. For the sake of figure clarity, individual data are shown only on one panel. Symbols: * P<0.05 – P=0.001 vs. respective vehicle (dose “0” mg/kg), # P<0.05 - P=0.001 vs respective group data at stimulus duration of 1s; Sidak post-hoc test. The number of animals tested is shown on the bottom of the top panel’s bars. Data represent mean + SEM. Yellow backgrounds indicate the effects of (R)-ketamine; blue backgrounds of (S)-ketamine.

In this set of analyses, we investigated whether there is an interaction between of (*R*)- or (*S*)-ketamine dose and stimulus duration (Sd 1s vs Sd 2s), thus analyses of variance were constructed to investigate only the interaction between these factors. For every measure two independent ANOVAs were performed: for (*R*)-ketamine and (*S*)-ketamine. In case of significant interaction, Sidak post-hoc test was performed.

*Accuracy*: For (*R*)-ketamine, ANOVA demonstrated significant interaction between the dose and stimulus duration (F(7,111)=2.907; P=0.008); the post-hoc test revealed no differences between various doses of (*R*)-ketamine and vehicle but significant differences between stimuli duration for vehicle and (*R*)-ketamine at 30 mg/kg (P<0.05). Also for (*S*)-ketamine, ANOVA demonstrated significant interaction between the dose and stimulus duration (F(7,122)=3.395; P=0.002; the post-hoc test revealed no effects among the doses but significant differences between stimuli duration for (*S*)-ketamine at 3.75 (P=0.023), 7.5 (P=0.027), and 15 mg/kg (P=0.001; Supplemental Figure 8**, top panel**).

*Correct responses*: For (*R*)-ketamine, ANOVA demonstrated significant interaction between the dose and stimulus duration (F(7,111)=3.931; P=0.001); the post-hoc test revealed a decrease of correct responses produced by 30 mg/kg dose (P=0.014) in 1s stimulus conditions and their *increase* in 2s stimulus duration conditions (P=0.001). For (*S*)-ketamine the interaction was also significant (F(7,122)=7.96; P<0.001) and doses of 7.5 and 15 mg/kg decreased correct responses (P<0.01) at Sd 1s, while 15 mg/kg of (*S*)-isomer decreased them at Sd 2s (P<0.01). Correct responses decrease produced by 7.5 mg/kg at Sd 1s was reversed in Sd 2s conditions (P=0.001; Supplemental Figure 8**, panel close to top**).

*Incorrect responses*: For (*R*)-ketamine, ANOVA demonstrated significant interaction between the dose and stimulus duration (F(7,111)=2.749; P=0.011); the post-hoc test revealed no effects of the dose on incorrect responses but their decrease in Sd 2s conditions for (*R*)-ketamine at 0 mg/kg (P=0.011). Also for (*S*)-ketamine the interaction was significant (F(7,122)=2.31; P<0.05); the post-hoc test revealed that increasing Sd to 2 s reduced incorrect responses for the dose of 3.75 mg/kg of (*S*)-isomer (Supplemental Figure 8**, panel close to bottom**).

*Omissions*: For (*R*)-ketamine, ANOVA demonstrated a significant interaction between the dose and stimulus duration (F(7,111)=5.115; P<0.001); the post-hoc test revealed an increase of omissions produced by 30 mg/kg dose (P<0.001) in Sd 1s conditions and their decrease in Sd 2s conditions (P=0.014). Also for (*S*)-ketamine, ANOVA demonstrated a significant interaction between the dose and stimulus duration (F(7,125)=9.319; P<0.001); doses of 7.5 and 15 mg/kg increased omissions (P<0.01) at Sd 1s, while 15 mg/kg of (*S*)-isomer increased omissions at Sd 2s (P<0.001). Omissions produced by 7.5 mg/kg at Sd 1s were decreased in Sd 2s conditions (P=0.001; Supplemental Figure 8**, bottom panel**).

## Effects of (*R*)- and (*S*)-ketamine on premature, perseverative and timeout responses in 5-CSRTT with different stimuli durations


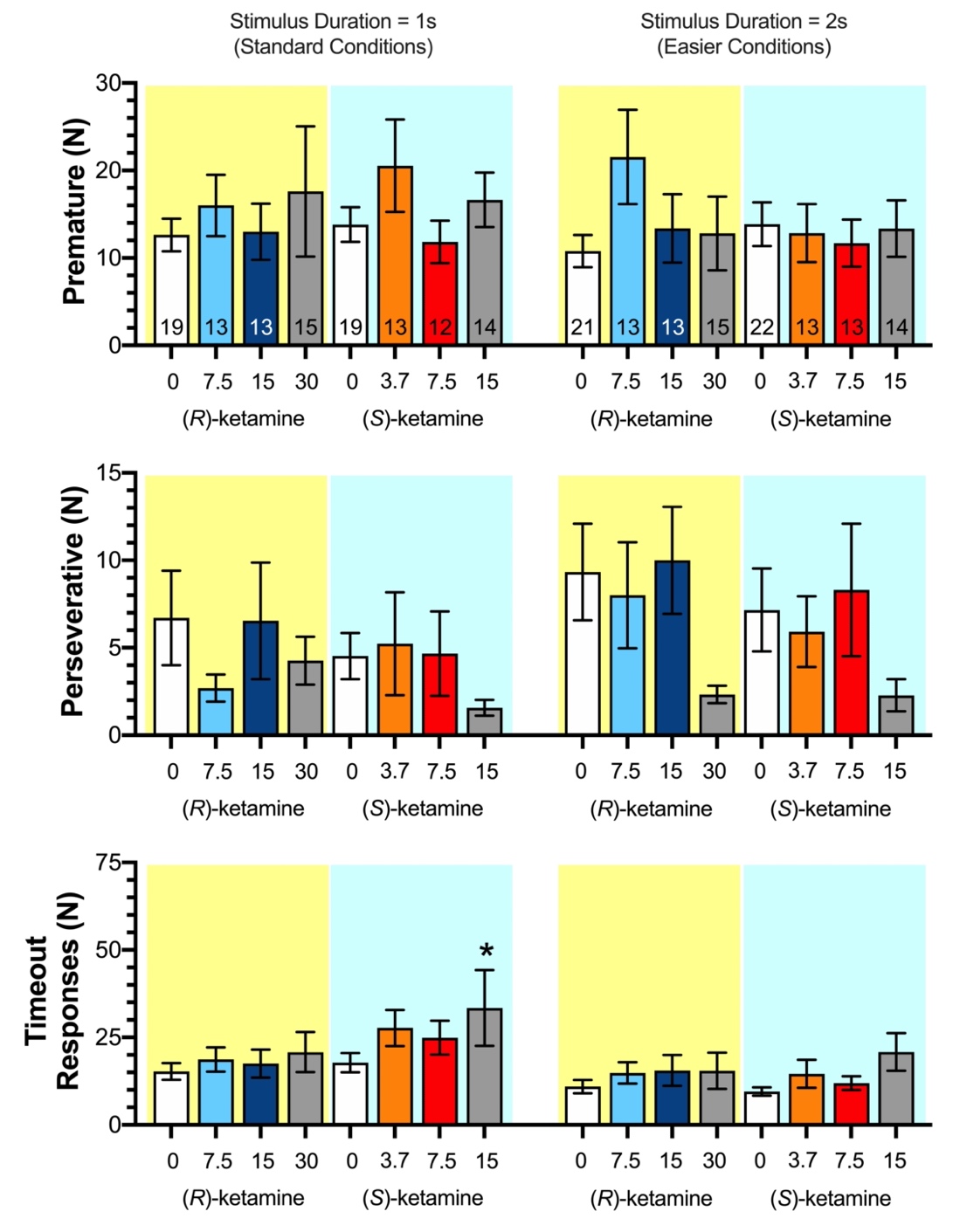


Supplemental Figure 9**.** The **top panel** shows that premature responses were unaltered by (R)- and (S)-ketamine and by stimulus duration, suggesting no effect on impulsivity-like behavior in the present experimental conditions. The lack of effect of ketamine doses and stimulus duration on perseverative responses may suggest no effects on compulsive-like behavior (**middle panel**). The **bottom panel** demonstrates increased timeout responses due to the highest dose of (S)-ketamine, suggesting some effects on compulsive-like behavior. Symbols: * P=0.031 vs. respective vehicle (dose “0” mg/kg); Sidak post-hoc test. Data represent mean + SEM. The number of animals is shown on the bottom of the top panel’s bars. Yellow backgrounds indicate the effects of (R)-ketamine; blue backgrounds of (S)-ketamine.

*Premature responses*: For neither (*R*)-ketamine (F(7,111)=0.775) nor for (*S*)-isomer (F(7,122)=0.883) ANOVA demonstrated significant interaction between the dose and stimulus duration (Supplemental Figure 9**, top panel**).

*Perseverative responses*: Neither for (*R*)-ketamine (F(7,111)=1.384) nor for (*S*)-ketamine (F(7,122)=1.138) ANOVA demonstrated significant interaction between the dose and stimulus duration (Supplemental Figure 9**, middle panel**).

*Timeout responses*: For (*R*)-ketamine, ANOVA demonstrated no significant interaction between the dose and stimulus duration (F(7,111)=0.594). For the (*S*)-isomer the interaction was significant (F(7,122)=3.341; P=0.003) and the dose of 15 mg/kg increased this measure in 1s Sd (P=0.031; Supplemental Figure 9**, lower panel)**

## Effects of (*R*)- and (*S*)-ketamine on latencies to correct and incorrect responses in 5-CSRTT with different stimuli durations


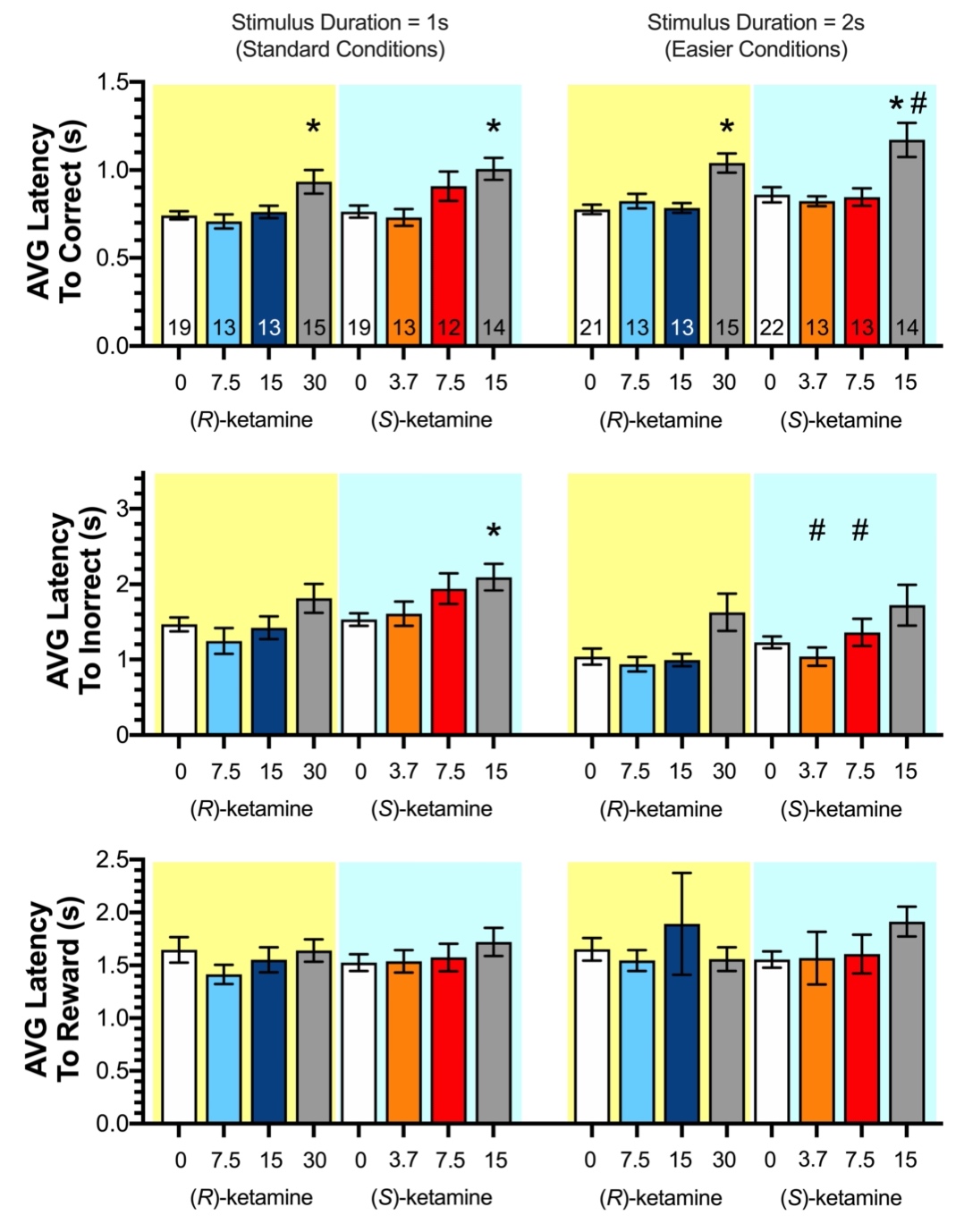


Supplemental Figure 10**.** The **top panel** shows latencies to correct responses that were prolonged by the highest doses of (R)- and (S)-ketamine and in the latter case by increased stimulus duration, suggesting unspecific behavioral effects. The **middle panel** shows latencies to incorrect responses that were increased by the highest dose of (S)-ketamine, but reduced in 2 s Sd conditions. The **bottom panel** demonstrates no effects of ketamine enantiomers on reward latency, suggesting no effect on motivation. Symbols: * P<0.05 - P<0.01 vs. respective vehicle (dose “0” mg/kg), # P<0.05 vs respective group data at stimulus duration of 1s; Sidak post-hoc test. Data represent mean + SEM. The number of animals tested is shown on the bottom of the top panel’s bars. Yellow backgrounds indicate the effects of (R)-ketamine; blue backgrounds of (S)-ketamine.

*Latency to correct responses*: For (*R*)-ketamine, ANOVA demonstrated significant interaction between the dose and stimulus duration (F(7,111)=6.242; P<0.001); the post-hoc test revealed increased latencies for 30 mg/kg of (*R*)-ketamine at Sd 1s and Sd 2s (P<0.05). Also for (*S*)-ketamine the interaction was significant (F(7,122)=7.178; P<0.001) and the dose of 15 mg/kg increased latencies at both stimuli durations (P<0.01). For the same dose, latencies to correct responses were further increased at Sd 2s (P<0.05; Supplemental Figure 10 **top panel**).

*Latency to incorrect responses*: For (*R*)-ketamine, ANOVA demonstrated significant interaction between the dose and stimulus duration (F(7,111)=4.095; P<0.001); the post-hoc test revealed no dose or Sd differences. Also for (*S*)-ketamine the interaction was significant (F(7,122)=5.129; P<0.001) and the dose of 15 mg/kg increased latencies at Sd 1s (P<0.01). Increasing Sd to 2 s reduced latency to incorrect responses for (*S*)-ketamine at doses of 3.75 and 7.5 mg/kg (P<0.05; Supplemental Figure 10 **middle panel**).

*Latency to reward*: Neither for (*R*)- nor for (S)-ketamine ANOVA showed significant interaction between the dose and stimulus duration (F(7,111)=0.551) and (F(7,122)=0.921), respectively (Supplemental Figure 10 **bottom panel**).

## Effects of psilocybin and psilocin on accuracy and correct and incorrect responses and on omissions in 5-CSRTT with different stimuli durations


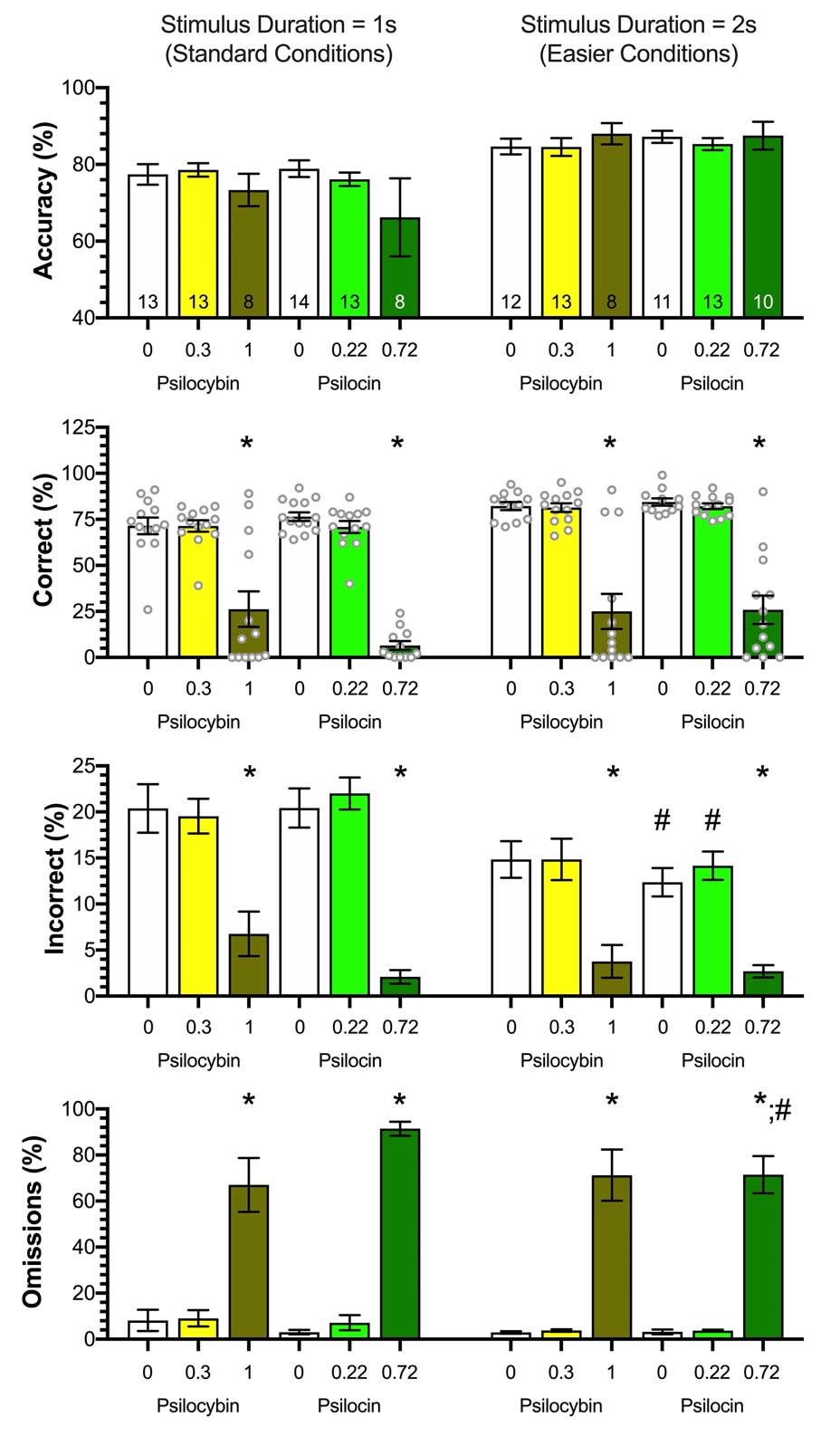


Supplemental Figure 11. The **top panel** shows no effects of psilocybin and psilocin on accuracy in 5-CSRTT with different stimulus duration conditions, but decreased correct responses for the highest doses of both compounds (panel **close to top**). The same doses reduced also incorrect responses, which in case of psilocin (0.22 mg/kg) and its vehicle, were reduced in Sd 2-s conditions (panel **close to bottom**). The highest doses of psilocybin and psilocin dramatically increased response omissions that were reduced for 0.72 mg/kg of psilocin in Sd 2-s conditions (**bottom panel**). Symbols: * P<0.05 vs. respective vehicle (dose “0” mg/kg); # P<0.05 vs. respective treatment at Sd 1-s; Sidak post-hoc test. Data represent mean + SEM. The number of animals tested is shown on the bottom of the top panel’s bars.

*Accuracy*: For psilocybin (F(2,61)=1.388) and psilocin (F(2,63)=1.826) ANOVA demonstrated no significant interaction between the dose and stimulus duration (Supplemental Figure 11**, top panel**).

*Correct responses*: For psilocybin, ANOVA demonstrated no significant interaction between the dose and stimulus duration (F(2,71)=0.592) but a significant dose factor (F(2,71)=45.461; P=0.001); the post-hoc test revealed a decrease of correct responses produced by 1 mg/kg dose (P<0.05) in 1-s and 2-s stimulus conditions. Also for psilocin the interaction was not significant (F(2,70)=1.108) though correct responses were affected by the dose (F(2,70)=168.975; P<0.001) and the post-hoc test showed their reduction by 0.72 mg/kg of psilocin (P<0.05; Supplemental Figure 11**, panel close to top**).

*Incorrect responses*: For psilocybin, ANOVA demonstrated no significant interaction between the dose and stimulus duration (F(2,71)= 0.176) but a significant dose factor (F(2,71)=20.632; P<0.001); the post-hoc test revealed a decrease of incorrect responses produced by 1 mg/kg dose (P<0.05) in 1-s and 2-s stimulus conditions. For psilocin, significant interaction was found (F(2,70)=5.200; P=0.008); the post-hoc test showed decreased incorrect responses for 0.72 mg/kg of psilocin at both Sd 1-s and 2-s conditions; however these produced by 0.22 mg/kg of psilocin and its vehicle at Sd 1-s were reduced in Sd 2-s conditions (P<0.05; Supplemental Figure 11**, panel close to bottom**).

*Omissions*: For psilocybin ANOVA showed no significant interaction between the dose and stimulus duration (F(2,71)=0.300), but a significant dose effect (F(2,71)=52.941; P<0.001); the post-hoc test revealed increased omissions for 1 mg/kg of psilocybin (P<0.05). In contrast, significant dose x Sd interaction was revealed for psilocin (F(2,70)=3.717; P<0.05) and the post-hoc test showed increased omissions for 0.72 mg/kg of psilocin in both Sd 1-s and 2-s conditions; however omissions produced by 0.72 mg/kg of psilocin at Sd 1-s were reduced in Sd 2-s conditions (P<0.05; Supplemental Figure 11**, bottom panel**).

## Effects of psilocybin and psilocin on premature, perseverative and timeout responses in 5-CSRTT with different stimuli durations


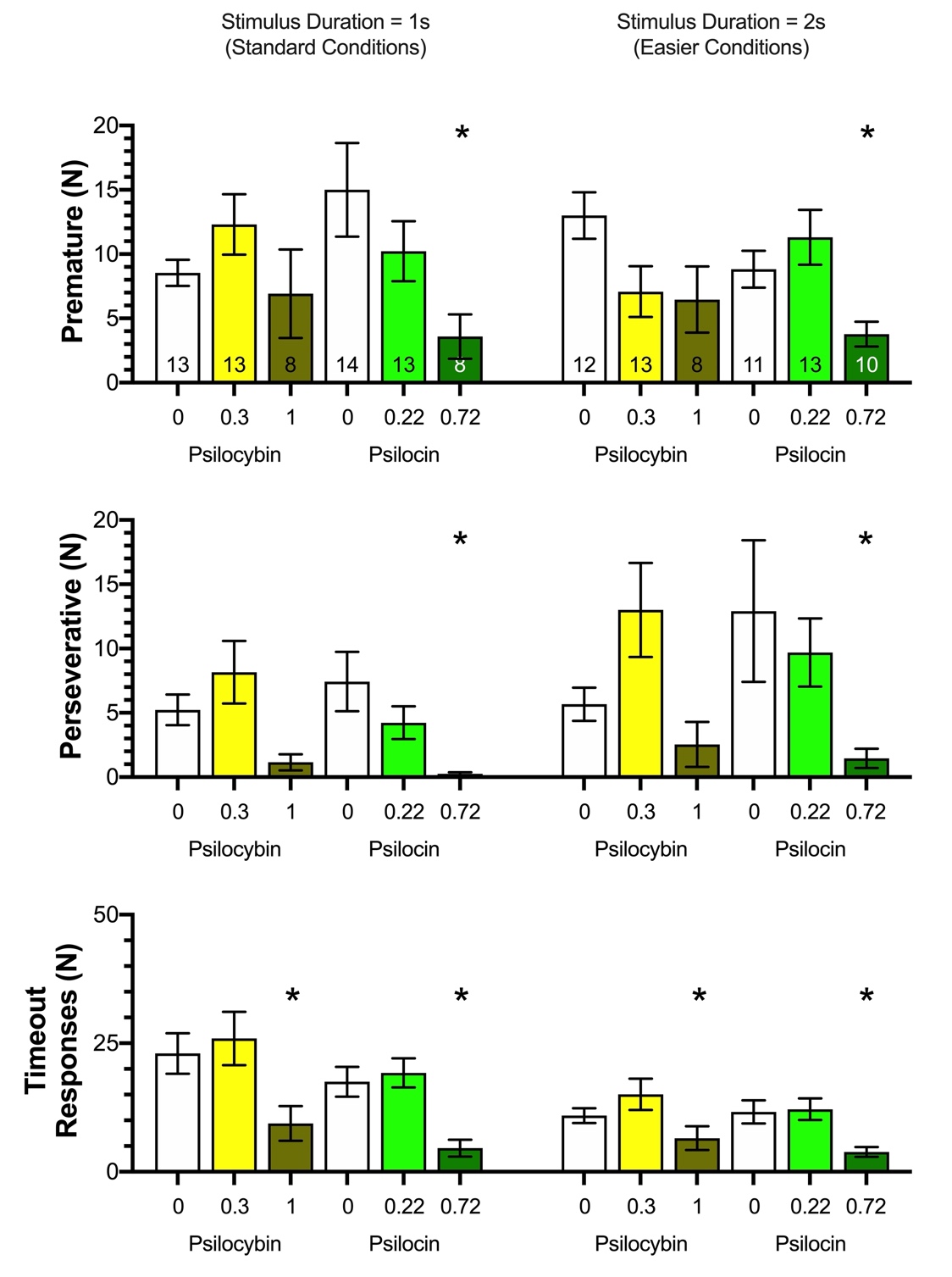


Supplemental Figure 12**.** The **top panel** shows that the premature responses were decreased by psilocin (0.72 mg/kg), suggesting reduction of impulsivity-like behavior in the present experimental conditions. The same dose of psilocin reduced also perseverative responses suggesting reduced compulsive-like behavior (**middle panel**). The **bottom panel** demonstrates decreased timeout responses due to the high doses of psilocybin and psilocin. Symbols: * P<0.05 vs. respective vehicle (dose “0” mg/kg); Sidak post-hoc test. Data represent mean + SEM. The number of animals tested is shown on the bottom of the top panel’s bars.

*Premature responses*: For psilocybin and psilocin ANOVA demonstrated no significant interaction between the dose and stimulus duration (F(2,71)=2.146) and F(2,70)=1.443); respectively. In addition, for psilocin the dose factor was significant (F(2,70)=7.329; P=0.001) and the post-hoc test showed decreased premature responses for the dose of 0.72 mg/kg (P<0.05; Supplemental Figure 12 **top panel).**

*Perseverative responses*: For psilocybin ANOVA demonstrated no significant interaction between the dose and stimulus duration (F(2,71)=0.617) but a significant effect of the dose (F(2,71)=8.923; P<0.001); the post-hoc test revealed a marginally significant *increase* in perseverations (P=0.05) due to 0.3 mg/kg of psilocybin. A similar pattern was revealed for psilocin: ANOVA demonstrated no significant interaction between the dose and stimulus duration (F(2,70)=0.462) but a significant effect of the dose (F(2,70)=6.775; P=0.002); the post-hoc test revealed a significant *decrease* in perseverations (P<0.05) due to 0.72 mg/kg of psilocin (Supplemental Figure 12**, middle panel**).

*Timeout responses*: For psilocybin ANOVA demonstrated no significant interaction between the dose and stimulus duration (F(2,71)=1.048) but a significant effect of the dose (F(2,71)=7.023; P=0.002); the post-hoc test demonstrated decreased timeout responses (P<0.05) due to 1 mg/kg of psilocybin. A similar pattern was found for psilocin: ANOVA demonstrated no significant interaction (F(2,70)=1.102) but a significant effect of the dose (F(2,70)=15.541; P<0.001); the post-hoc test revealed a significant decrease in perseverations (P<0.05) due to 0.72 mg/kg of psilocin (Supplemental Figure 12**, lower panel)**

## Effects of psilocybin and psilocin on latencies to correct and incorrect responses in 5-CSRTT with different stimuli durations


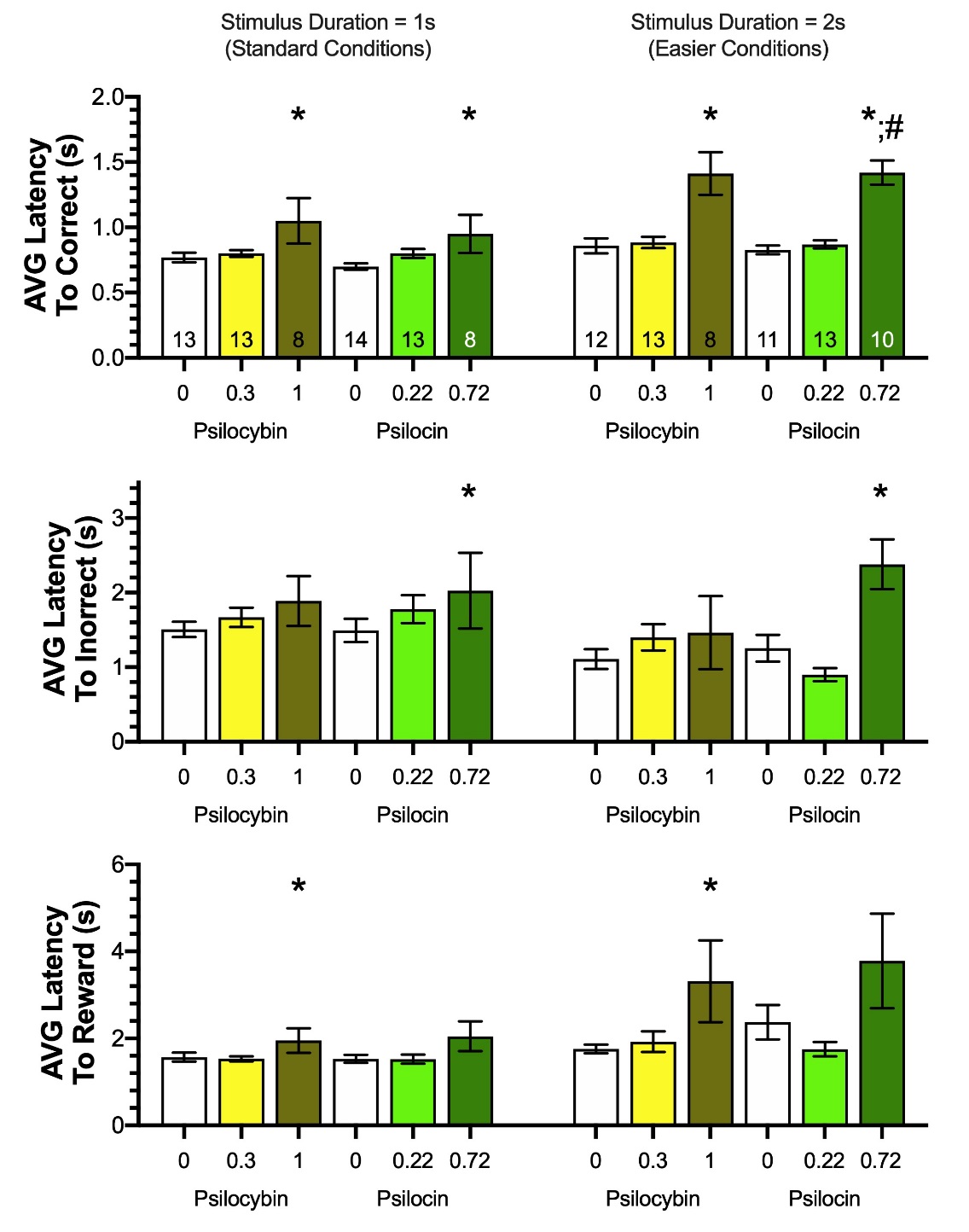


Supplemental Figure 13**.** The **top panel** shows latencies to correct responses that were prolonged by the highest doses of psilocybin and psilocin; in the case of 0.72 mg/kg of psilocin they were further increased in Sd 2-s conditions, suggesting unspecific behavioral effects. The **middle panel** shows latencies to incorrect responses that were increased by the highest dose of psilocin in both stimulus duration conditions. The **bottom panel** demonstrates increased reward latencies produced by highest doses of either compounds, which in case of psilocybin were statistically significant, suggesting unspecific effects or impaired motivation. Symbols: * P<0.05 vs. respective vehicle (dose “0” mg/kg), # P<0.05 vs respective group data at stimulus duration of 1s; Sidak post-hoc test. Data represent mean + SEM. The number of animals tested is shown on the bottom of the top panel’s bars.

*Latency to correct responses*: For psilocybin ANOVA demonstrated no significant interaction between the dose and stimulus duration (F(2,71)=1.731) but a significant effect of the dose (F(2,71)=14.653; P<0.001); the post-hoc test revealed significant increase in latency to correct responses (P<0.05) due to 1 mg/kg of psilocybin. In case of psilocin ANOVA showed significant interaction (F(2,63)=6.303; P=0.003); the post-hoc test demonstrated that latencies to correct responses that were prolonged by 0.72 mg/kg of psilocin and were further increased in Sd 2-s conditions (P<0.05; Supplemental Figure 13**, top panel).**

*Latency to incorrect responses*: For psilocybin, ANOVA demonstrated no significant interaction between the dose and stimulus duration (F(2,61)=0.077). For psilocin ANOVA showed marginally significant interaction (F(2,63)=3.143; P=0.05) but a significant dose factor (F(2,63)=7.813; P=0.001); the post-hoc test showed increased latencies for incorrect responses due to 0.72 mg/kg psilocin (P<0.05; Supplemental Figure 13**, middle panel).**

*Latency to reward*: For psilocybin ANOVA showed no significant interaction between the dose and stimulus duration (F(2,61)=1.711), but a significant effect of the dose (F(2,61)= 4.955; P=0.01); the post-hoc test revealed a significant increase in latency to reward (P<0.05) due to 1 mg/kg of psilocybin. A similar pattern was found for psilocin: no significant interaction (F(2,63)=1.334) and a significant dose effect (F(2,63)=4.148; P=0.02); however for psilocin the post-hoc test revealed no significant effects vs. vehicle (Supplemental Figure 13**, bottom panel).**

## Effects of norpsilocin in 5-CSRTT with standard 1 s stimulus duration conditions


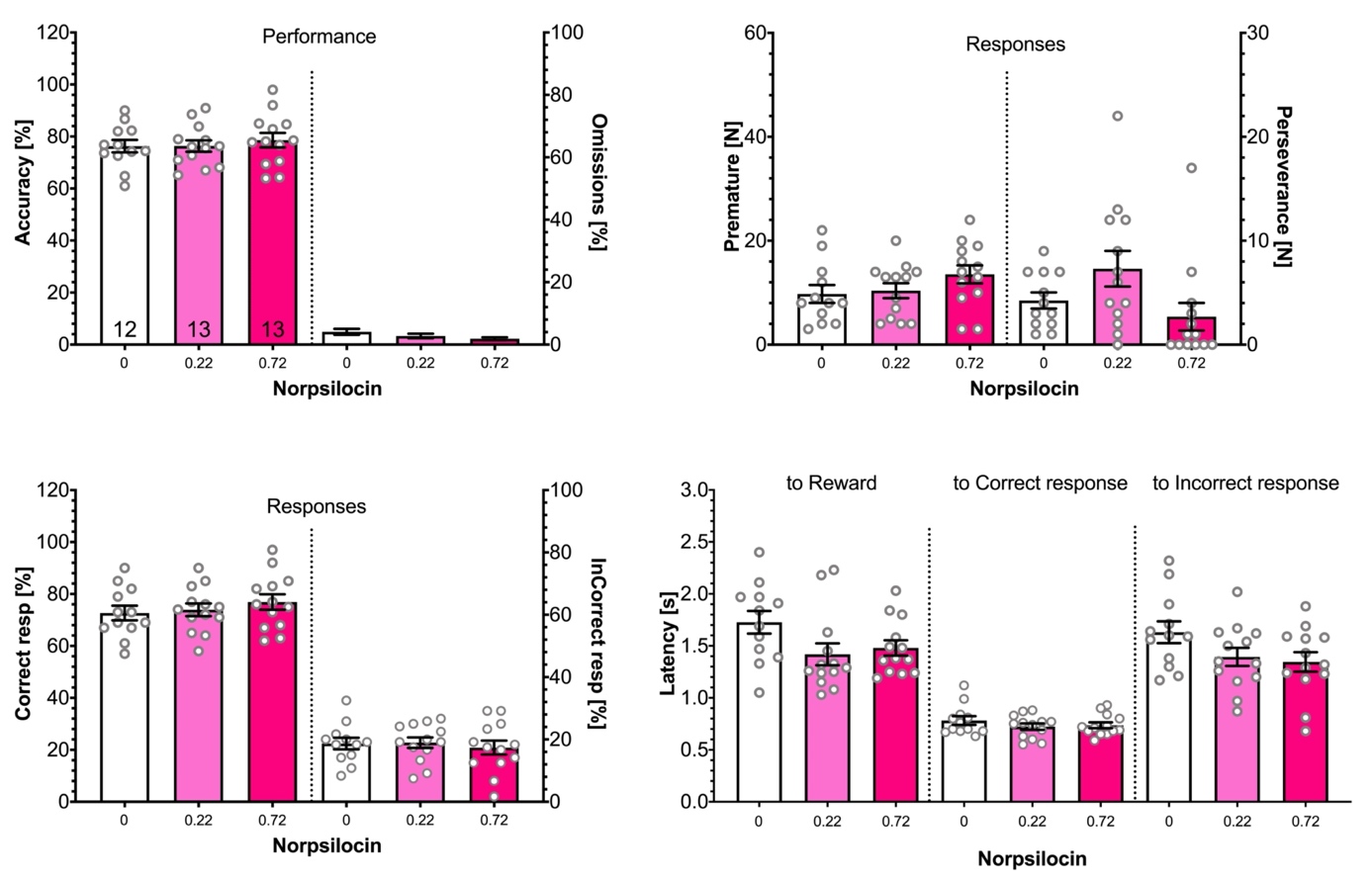


Supplemental Figure 14. No effects of norpsilocin in 5-CSRTT in the standard stimulus duration conditions. Data represent mean + SEM. The number of animals tested is shown on the bottom of the upper left panel’s bars.

One way ANOVA demonstrated no effects of norpsilocin on accuracy and omissions (F(2,35)<1) and (F(2,35)=2.296), respectively; Supplemental Figure 14 **upper left panel**, premature and perseverative responses (F(2,35)=1.546) and (F(2,35)=3.077; P=0.059), respectively; Supplemental Figure 14 **right panel**, correct and incorrect responses (F(2,35)<1); Supplemental Figure 14 **lower left panel**, latency to correct response (F(2,35)<1), to incorrect response (F(2,35)=2.533) and reward latency (F(2,35)=2.763; Supplemental Figure 14 **lower right panel**) in the standard Sd 1-s 5-CSRTT conditions.

## Effects of (*R*)- and (*S*)-ketamine on latencies to correct and incorrect responses and on reward latencies in 5-CSRTT in variable ITI conditions


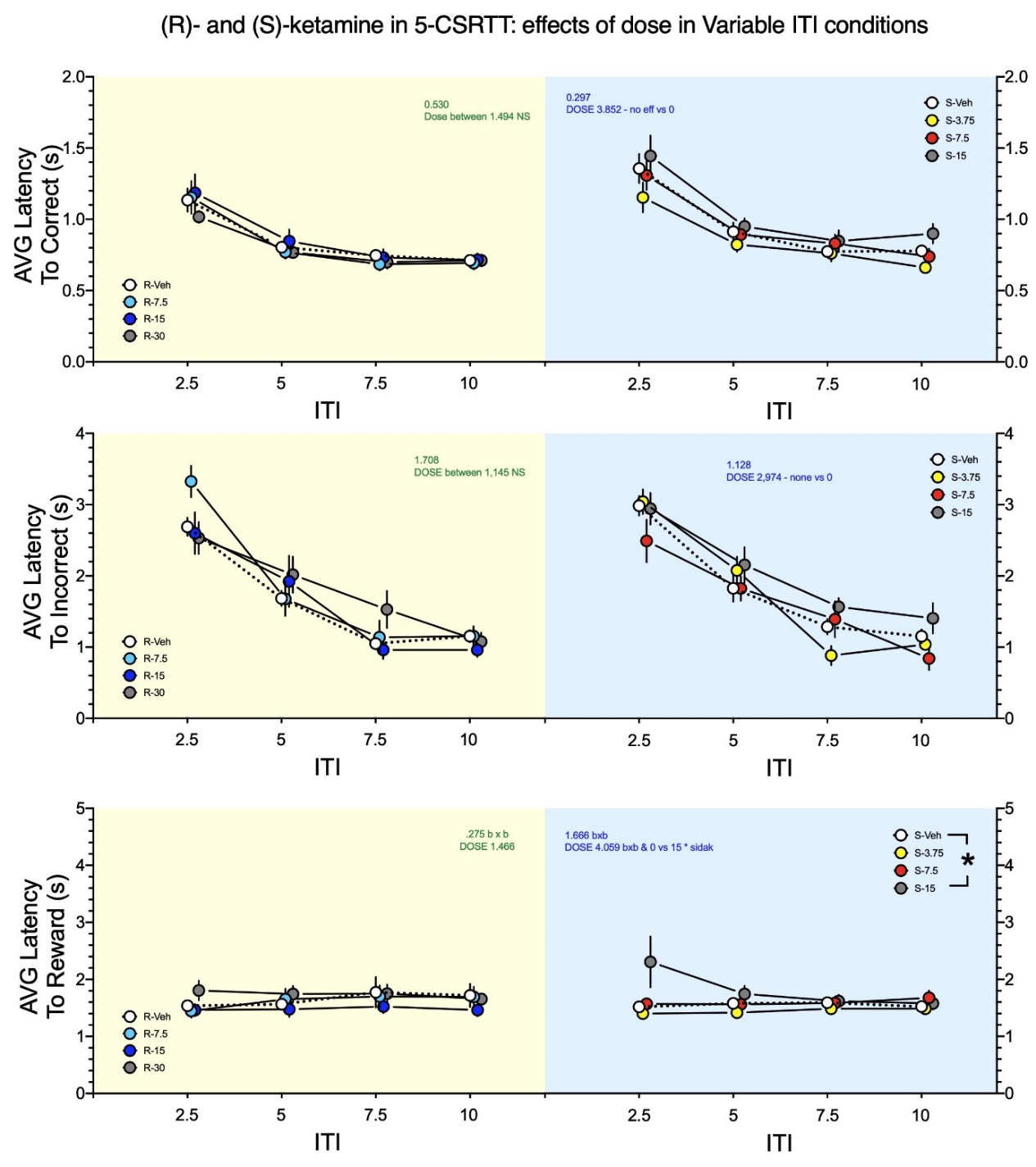


Supplemental Figure 15**.** The **top and middle panels** show no effects of ketamine enantiomers on latencies to correct and incorrect responses, respectively. The **bottom panel** demonstrates that while (R)-ketamine did not affect latency to reward, (S)-ketamine at the highest dose increased it (*, P<0.05), suggesting unspecific effects of this isomer. Data represent mean + SEM. Yellow backgrounds indicate the effects of (R)-ketamine; blue backgrounds of (S)-ketamine.

*Latency to correct response*: For (*R*)-ketamine ANOVA showed neither significant interaction between the dose and ITI (F(9,292)=0.530) nor the dose factor (F(3,292)=1.494). For (*S*)-ketamine ANOVA showed no significant interaction between the dose and ITI (F(9,275)=0.297) but a significant dose factor (F(3,275)=3.852; P=0.008); however, the post-hoc test showed no differences vs vehicle (Supplemental Figure 15**, top panel**).

*Latency to incorrect response*: For (*R*)-ketamine ANOVA showed neither significant interaction between the dose and ITI (F(9,292)=1.708) nor the dose factor (F(3,292)=1.145). For (*S*)-ketamine ANOVA showed no significant interaction between the dose and ITI (F(9,275)=1.128) but a significant dose factor (F(3,275)=2.974; P=0.008); the post-hoc test showed, however, no differences vs vehicle (Supplemental Figure 15**, middle panel**).

*Latency to reward*: For (*R*)-ketamine ANOVA showed neither significant interaction between the dose and ITI (F(9,292)=0.275) nor the dose factor (F(3,292)=1.466). For (*S*)-ketamine ANOVA showed no significant interaction between the dose and ITI (F(9,275)=1.666) but significant dose factor (F(3,275)=4.059; P=0.008); the post-hoc test showed increased reward latencies for the dose of 15 mg/kg (P<0.05; Supplemental Figure 15**, bottom panel**).
